# LDscaff: LD-based scaffolding of de novo genome assemblies

**DOI:** 10.1186/s12859-020-03895-7

**Published:** 2020-12-28

**Authors:** Zicheng Zhao, Yingxiao Zhou, Shuai Wang, Xiuqing Zhang, Changfa Wang, Shuaicheng Li

**Affiliations:** 1 BGI Education Center, University of Chinese Academy of Sciences, Shenzhen, 518083 China; 2 Department of Computer Science, City University of Hong Kong, Kowloon, Hong Kong SAR 999077 China; 3 Liaocheng Research Institute of Donkey High-Efficiency Breeding and Ecological Feeding, Liaocheng University, Liaocheng City, 252059 Shandong China; 4 BGI–Shenzhen, Shenzhen, 518083 China

**Keywords:** *De novo* assembly, Maximum weighted matching, Linkage disequilibrium

## Abstract

**Background:**

Genome assembly is fundamental for *de novo* genome analysis. Hybrid assembly, utilizing various sequencing technologies increases both contiguity and accuracy. While such approaches require extra costly sequencing efforts, the information provided millions of existed whole-genome sequencing data have not been fully utilized to resolve the task of scaffolding. Genetic recombination patterns in population data indicate non-random association among alleles at different loci, can provide physical distance signals to guide scaffolding.

**Results:**

In this paper, we propose *LDscaff* for draft genome assembly incorporating linkage disequilibrium information in population data. We evaluated the performance of our method with both simulated data and real data. We simulated scaffolds by splitting the pig reference genome and reassembled them. Gaps between scaffolds were introduced ranging from 0 to 100 KB. The genome misassembly rate is 2.43% when there is no gap. Then we implemented our method to refine the Giant Panda genome and the donkey genome, which are purely assembled by NGS data. After *LDscaff* treatment, the resulting Panda assembly has scaffold N50 of 3.6 MB, 2.5 times larger than the original N50 (1.3 MB). The re-assembled donkey assembly has an improved N50 length of 32.1 MB from 23.8 MB.

**Conclusions:**

Our method effectively improves the assemblies with existed re-sequencing data, and is an potential alternative to the existing assemblers required for the collection of new data.

## Background

With the massive increases in the throughput of the Next Generation Sequencing (NGS) technique, a large number of organisms have been sequenced and assembled [1–8]. Most current assembly approaches stitched short reads together to generate contigs and scaffolds. Though NGS provides accurate base-level sequences, specific regions such as nonrandom repeat elements can hardly be accurately assembled. The reason for the contiguity problem is typical short reads with lengths in the range of 20–500 bp can hardly cover the repeat regions  [9–11]. Also, vulnerable spots that may introduce gaps in the assembly process [9, 11, 12] due to uneven sequencing coverage.

Long-range scaffolding technologies can provide long-range connectivity, which can also aid in resolving the complex regions. Such methods include end sequencing of fosmid clones [1], fosmid-based dilution pool sequencing [13, 14], optical mapping [15–17], genetic mapping with restriction site associated DNA (RAD) tags [18] and proximity ligation (Hi-C) sequencing. However, each of these methods has a limitation in either experimental cost or application scenarios [19]. Fosmid cloning is sensitive to the quantity and quality of the input DNA, while fosmid libraries are subject to cloning bias. The data generating process for optical map construction involves mostly manual steps. These steps include DNA extension and image capture, which are low throughput and inefficient. Genetic maps are costly or impractical to generate from many species. Although the Hi-C data provide extensive links covering large distances, the current resolution is not high enough for the local ordering of small adjacent contigs.

Linkage disequilibrium(LD) is the non-random association of alleles at different loci in population genetics [20, 21]. LD is of importance in population genetics because it reflects evolutionary history. It is derived from several population genetic forces that structure a genome, such as population selection, recombination, mutation, genetic drift, mating rate, population sex ratio, and genetic linkage. Genetic linkage maps constructed from population data now provide the basis for a wide range of genomics studies. LD depends strongly on one-dimensional distance and can extend over 550 KB [22]. LODE [23] uses this kind of linkage information to place unpositioned SNPs by estimating LD with SNPs with confirmed locations. LD maps constructed from SNP data can guide the ordering of contigs from a 216 KB region [24]. Thus, the high-density inter-marker LD in the population dataset has the potential to inform the orders and orientation of scaffolds over a large distance. Some methods have been published to integrate whole genome sequencing(WGS) data and linkage map construction. POPSEQ [25] requires samples from a known crossing design to assemble a barley genome. Recombinant Population Genome Construction [26] first build a ‘consensus’ assembly from sequencing a population of recombinant individuals, then a linkage map was generated to improve the assembly. The joint assembly and mapping method [27] constructs a high-density genetic map to exam the genome organization. Either these methods require specific crossing designs or a built linkage map. According to our best knowledge, there is no available tool to guide scaffolding based on LD without building linkage maps.

Population analysis is essential in species genome studies to investigate the structural and variants among individuals as well as their evolutionary history. Currently, a large number of draft genomes in NCBI are assembled purely by short sequence reads. Accompanied by these draft genomes, whole-genome resequencing data have also experienced rapid development but have not obtained considerable integration and manipulation. Here we present *LDscaff* to consider whether linkage information obtained from single-nucleotide variations in population, combined with short reads data, is capable of providing extra information in scaffold contiguity. *LDscaff* aids in the layout of a set of scaffolds with a graph method, by taking as input the population variation data, a set of scaffolds, to build an undirected graph with a set of vertices and edges, representing the scaffolds and the LD strength among them. Computing the optimal orders and orientations of these scaffolds can be treated as a maximum weight matching problem.

We applied our method to both simulated data and empirical data to verify the effectiveness of our method. The simulation experiments were performed on a pig genome. We randomly split the pig reference genome [28] into 360 scaffolds and tried to reassemble them. The average error rate (percentage of misassemblies) is 2.43% in 20 experiment trials. We then refined the draft Giant Panda genome [4] and a *de novo* donkey genome assembly. These draft assemblies were both assembled using only short reads. The resulting Giant Panda assembly has a scaffold N50 of 3.6 MB, 2.5 folders larger than the original one. The re-assembled donkey assembly has an improved N50 length of 32.1 MB.

## Implementation

### The scaffolding problem

The principle of whole-genome shotgun assemblies is to assign, order, and orient sequence contigs. Our method solves the scaffolding problem with a graphical algorithm. We build a complete graph *G*, with vertices *V* representing scaffolds, and edge weights *E* corresponding to the linkage power between pairs of scaffolds. Given a weighted graph *G*, the problem is transformed into finding a set of edges that have the maximum sum-up weight and do not share common vertices. The problem is known as the maximum matching problem in graphical theory.

### Data prepossessing

We downloaded the giant panda reference AilMel 1.0 from the NCBI GenBank database (Accession number: GCA000004335.1). The genome coverage is 60x, and the N50 of contigs and scaffolds are 39,886 bp and 1,281,781 bp respectively. We downloaded the chromosome-level panda genome [29] from the National Genomics Data Center (Accession Number: GWHACDL00000000). The genome coverage is 82x, and the genome sequence N50 is 129,245,720 bp.

A purebred donkey individual was sequenced on an Illumina HiSeq 2000 sequencing platform. The paired-end reads were initially assembled with SOAPdenovo v2.04.4 [30] to construct short but accurate scaffolds. Tiny scaffolds shorter than 2 KB (containing 5.45% sequence bases) were set aside for insufficient linkage signal, and 2974 scaffolds remained. The assembly contained 2.4 Gb of sequence (scaffold N50: 22.0 MB).

We downloaded the panda population data from the NCBI Short Read Archive (SRA) database under accession SRA053353. In total, we obtained 34 panda samples, and then we aligned them to the reference with the BWA aln algorithm (Version 0.7.13) [31]. The bam files were converted and sorted using Samtools (Version 1.3.1) [32]. We used Freebayes (Version 0.9.10) [33] to call SNPs with default parameters. After that, we used GATK VariantFiltration [34] to extract reliable variants with strict criteria ($QD< 2.0, FS > 60.0, MQ< 40.0, MQRankSum< 12.5, ReadPosRankSum < 8.0$). Sorted BAM files were recalibrated with GATK BQSR with reliable SNPs. Local realignment around indels was performed with reliable indels using GATK Indel Realigner. SNPs were called and filtered using GATK HaplotypeCaller with the same strict criteria as above. In total, we obtained 13 million (13,427,006) SNPs.

Population resequencing data from 132 donkeys were collected. These samples were sequenced on an HiSeq 4000 platform. Trimmomatic [35] was used to remove adaptor contamination. Cleaned reads were mapped to the donkey assembly using BWA (version: 0.7.10-r789). High-quality reads were selected ($MQ>20$) with SAMTools (version 1.3.1). Regions covered by at least two reads in most samples (80%) were extracted. Duplications were marked and removed with Picard. High confident SNPs were obtained after GATK HaplotypeCaller and hard filtering were conducted ($QD< 2.0, FS > 60.0, MQ< 40.0, MQRankSum < 12.5$). Finally, the original BAM files were recalibrated with GATK BQSR with this set of SNPs. Among final SNPs, variants with small minor allele frequency less than 0.2 were filtered out.

### Building scaffolding graph

**Fig. 1:**
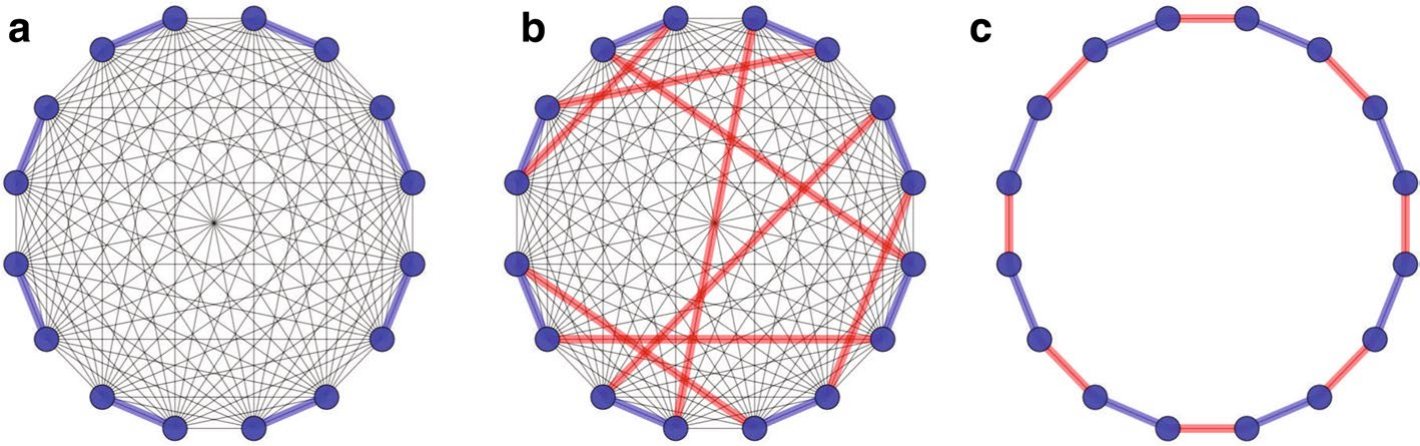
Illustration of the algorithm. **a** A complete graph constructed from eight scaffolds. The blue lines are the inner-edges that represent the vertexes belong to the same scaffold. The black lines are the outer-edges that represent the linkage between vertexes that are in the different scaffolds; **b** The set of red lines is a solution of the matching problem for the complete graph in **a**. All the red lines do not share comm-on vertexes; **c** The solution of the matching problem (red lines) and the inner-edges (blue lines) can form a circle. The linear permutation and the orientation of the scaff-olds can be obtained by remove the red line with the lowest linkage signal

Denote the graph G with the vertex set V and edge set E, each vertex refers to one side of a scaffold. For n scaffolds, the number of vertices in G is 2n. Edges are added for any two vertices. The graph is shown in Fig. 1a. We classify the edges into two groups, inner-edges $E_i$, and outer-edges $E_o$. An inner-edge (blue lines in Fig. 1a) connects two vertices that belong to the same scaffold, while an outer-edge (black lines in Fig. 1a) does not. The weight of an outer-edge is the linkage power between the two corresponding vertices. The inner–edges have weights of zero.

**Fig. 2:**
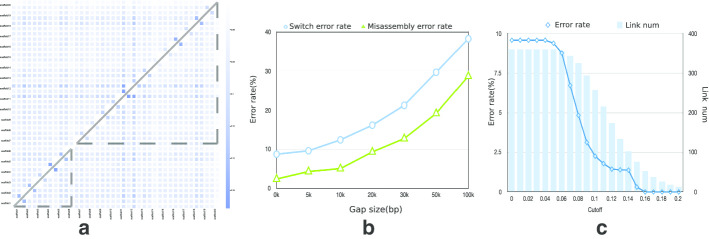
LDscaff performance in simulated data. **a** The heatmap for the LD ma-trix in the simulated experiment with 40 vertices in 20 scaffolds, pixel intensity in the matrix indicates the strength of LD. Solid lines represent correct scaffolding orders, while dash lines represent misassembly; **b** The error rates versus gap sizes; **c** The error rates and numbers of matching links using different cutoff thresholds

### Linkage power calculation

Each outer-edge connects two distinct vertices, referring to two scaffold ends. For each scaffold end, m markers (m = 100 by default) were extracted. Two-loci LD is calculated for m*m marker pairs. Linkage power for two scaffold ends is the average value for all pairwise LD statistics. Short scaffolds (with markers fewer than m) were set aside for insufficiency to provide linkage information.

The linkage power between the two sides can be considered as the approximation of the physical distance between the vertices—the larger the linkage power, the shorter physical distances on the genome. In reality, the inference of distance proximity from residue pair linkage is susceptible to both false negative and false positives. In our cases, allele distributions are estimated from a finite sample. Thus, a spurious nonzero allele frequency is likely to contribute a large LD value. To solve this issue, we estimated the LD using balanced allele distribution.

Allele frequencies are balanced as follows. Consider two positions having two possible alleles, with alleles *A*, *a* at the first site and *B*, *b* at second site respectively, there are 9 possible genotype combinations. We use $k_i$ to denote the occupancy of each possible genotype. We also define $d_i$ as the number of samples to remove. All possible combinations are displayed in Table 1.

**Table 1:** Enumeration of the genotypes and the notations

Locus 1	Locus 2	Observed individual number	Withdraw individual number
AA	BB	$k_1$	$d_1$
AA	Bb	$k_2$	$d_2$
AA	bb	$k_3$	$d_3$
Aa	BB	$k_4$	$d_4$
Aa	Bb	$k_5$	$d_5$
Aa	bb	$k_6$	$d_6$
aa	BB	$k_7$	$d_7$
aa	Bb	$k_8$	$d_8$
aa	bb	$k_9$	$d_9$

**Table 2:** Comparison of the refined panda genome with SOAPdenovo-assembled genome

Cutoff	Scaffolds before	Scaffolds after	N50 before	N50 after
0.1	57	57	1335486	1158329675
0.15	57	254	1335486	20717847
0.2	57	1344	1335486	3358889
0.25	57	2123	1335486	1962870
0.3	57	2509	1335486	1625199
0.4	57	2843	1335486	1413235
0.5	57	2953	1335486	1353331
0.6	57	2981	1335486	1336590
0.7	57	2983	1335486	1335486

Allele frequencies for allele *A* and for allele *B* are introduced,

1 $$\begin{aligned} f_A= & {} \frac{2 \sum _{i \in \{1, 2, 3\}}{(k_i-d_i)} + \sum _{i \in \{4, 5, 6\}}{(k_i-d_i)}}{2 \sum _{i=1}^{9}{(k_i-d_i)}} \end{aligned}$$

2 $$\begin{aligned} f_B= & {} \frac{2 \sum _{i \in \{1, 4, 7\}}{(k_i-d_i)} + \sum _{i \in \{2, 5, 8\}}{(k_i-d_i)}}{2 \sum _{i=1}^{9}{(k_i-d_i)}} \end{aligned}$$

We expect the allele frequency of allele *A* and allele *B* are approximate to 0.5 with the minimum number of removed individuals. We balance the frequencies using integer linear programming.

$$\begin{aligned}&\min {\sum {d_i}}&\\ {\text {subject to}}&\\&\left| f_A - 0.5 \right| \leqslant \theta \\&\left| f_B - 0.5 \right| \leqslant \theta \\&d_i \ge 0, \forall i&\end{aligned}$$

The linkage disequilibrium denoted as $D_p$ is then calculated by the Fisher’s exact test with the balanced allele frequencies.

### Permutation and orientation

The solution for maximum matching in *G* indicates the orders and orientation of the scaffolds. The maximum matching only includes outer-edges, and the degree of each vertex equals one as shown in Fig. 1. The matching constructed by $E_i$ will form one or several circles in *G* with all the vertices in them (Fig. 1). Each circle can be transformed into a linear path by removing the outer edge with the weakest weight in $e_M$.

**Fig. 3:**
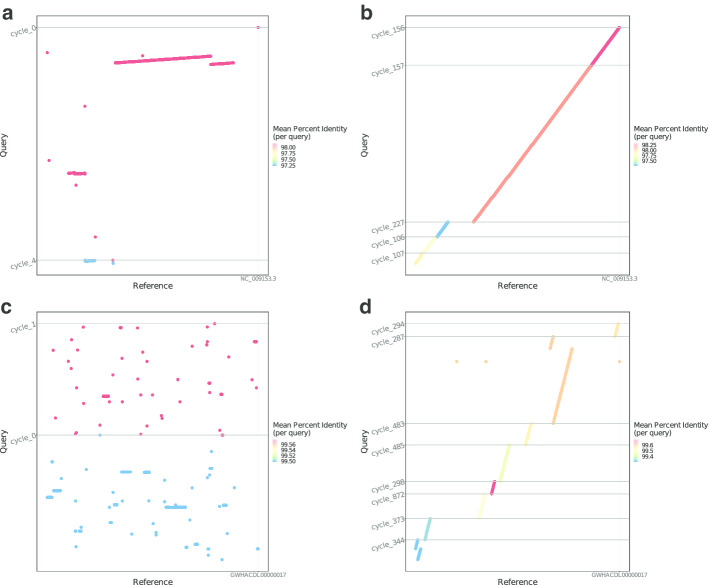
Dot-plots showing alignments of re-assembled scaffolds by ***LDscaff*** versus chromosome-length scaffolds. Cutoff of 0.1 was used in **a** and **c**, cutoff of 0.2 was used in **b** and **d**. The panda reference chr17 and the horse reference chr10 are shown on the X axis in upper part and lower part, respectively. The Y axis shows the re-assembled scaffolds (alignment longer than 5000bp, percentage of match higher than 70%). They have been ordered and oriented to match the chromosomes as defined in the reference in order to facilitate comparison. Each dot represents the position of an individual resolved scaffold aligned to the reference. The color of the dots and lines were colored to represent the match rate for each scaffold (red indicate higher mat-ching rate, whereas blue indicates lower matching rate)

**Fig. 4:**
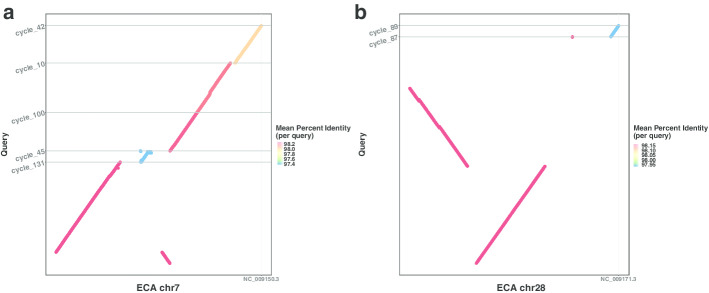
Chromosomal inversions were identified using LDscaff **a** A inversion mapped to horse reference chr7; **b** A inversion mapped to horse reference chr28

### Software implementation

There are several algorithms for solving the maximum matching problem in a general graph [36–38]. In *LDscaff* we applied Edmond’s blossom shrinking algorithm implemented by *lemon* [39]—an open-source graph library written in the C++ language.

### Evaluating assemblies

To evaluate the quality of assemblies, we mapped all assemblies to the corresponding reference genomes with nucmer [40] using the default parameters. We used QUAST [41] to collect various metrics (command line: “–eukaryote –min-contig 3000 –min-alignment 500 –extensive-mis-size 7000 –fast –split-scaffolds”).

## Results

We performed experiments on both simulated data and empirical data to evaluate the accuracy and effectiveness of our method.

### Simulation result

We simulated scaffolds by randomly splitting the pig assembly Sus scrofa 10.2 and applied *LDscaff* on the generated scaffolds to check whether it can produce the correct orders and orientations. For better illustration, we first select chr1 in Sus scrofa 10.2, and simulated input scaffolds by splitting the chromosome into 20 scaffolds. We created a complete graph with 40 vertices as demonstrated in the “Method” section. Linkage strength between any scaffold ends is calculated using 100 SNPs. The heatmap in Fig. 2a shows the corresponding LD matrix of scaffold end pairs. The vertices were labeled by the scaffold position order in reference. Orders of the vertices represent a permutation of the original scaffold ordering. Pixel intensity in the matrix indicates the strength of LD.

There are two patterns as showed in the heatmap. The first pattern is that the LD value between scaffold end pairs decay as their genomic distance increases. Adjacent vertices tend to have higher weights of LD. The second is that scaffolds from different chromosomes can be clustered utilizing the boundaries of the heatmap.

In Fig. 2a, solid lines that link diagonal white boxes imply correct scaffolding orders. One misassembly was outlined with two sets of dashed lines, one links scaffold1 and scaffold6 together, the other one links scaffold7 and scaffold20 together. We denoted the error rate as the percentage of misjoins of all scaffolds, evaluating the accuracy of *LDscaff*. The error rate, in this case, is 1/40 = 2.5%. We also extended the experiments to the whole genome-wide, we split the 18 chromosomes in the of Sus scrofa 10.2 by randomly splitting the 18 chromosomes into 360 scaffolds. We then built a graph and resolve the layout of the simulated scaffolds. With 20 experimental repeats, the average error rate is 2.43%.

As the LD linkage disequilibrium decays with the increase of the physical distance of the loci in the genome, we test how the performance of scaffolding is affected by gap sizes between the simulated scaffolds. We introduce gaps ranging from 5 to 100 KB respectively. To estimate the accuracy better, we introduced the switch error rate, which is the number of switches that required for transforming the solution into the correct matching divided by the total number of links. The error rates and the switch error rates for different gap sizes are shown in Fig. 2b. As expected, both kinds of error rates increase as the gap size increases. When the gap size becomes larger, false-positive signals tend to increase. Different cutoff thresholds to break to weakest links were also tested as shown in Fig. 2c.

### The giant panda genome

Based on the previously published panda assembly AilMel 1.0 purely from short reads, and a chromosome-level reference genome assembly (GWHACDL00000000) using linked-reads, we were able to test the accuracy of scaffolding. The AilMel 1.0 assembly was refined with *LDscaff*. After filtering, 2983 contigs remained and were linked into sets of cycles using different cutoff thresholds, as shown in Table 2. We then compared the generated sets of giant panda scaffolds with the GWHACDL00000000 assembly using mummer 3.23 [42] as shown in Fig. 2a, b using cutoff of 0.1 and 0.2, respectively. The complete alignment results with cutoffs 0.1, 0.2, and 0.3 are shown in Additional file 1: Figs. S1–S22. We broke the links that have LD weight less than 0.2 (termed as the cutoff), we get improved N50 (3.6 MB) 2.5 folder larger than the original one (1.3 MB) as shown in Table 2. Qualities of AilMel 1.0 assembly and refined assembly were evaluated using QUAST, the metric was shown in Table 3.

### The donkey genome

We applied our approach to improving a donkey genome assembly. We calculated scaffold pairwise LD strength with 100 SNPs at each side of the scaffold, scaffolds that not long enough to provide sufficient SNPs were filtered out. We then link the remaining 551 scaffolds using linkage information. The raw resulting assembly consisted of 80 cycles, they were then partitioned into 382 scaffolds (N50 length of 32.1 MB) after breaking the links with a cutoff of 0.2. The assembly now contains 94.3% of the total sequence (2.27G). The relationship of different cutoff thresholds and N50 of the refined assembly is shown in Table 4. The misassembly evaluation comparison metric of draft assembly and the refined assembly (cutoff equals 0.2) is shown in Table 5.

**Table 3:** Mis-assembly comparison of the refined panda genome with SOAPdenovo-assembled genome

Assembly evaluation	Before LDscaff	After LDscaff
Misassemblies	1092	448
Contig misassemblies	866	314
c. relocations	822	283
c. translocations	38	28
c. inversions	6	3
Scaffold misassemblies	226	134
s. relocations	220	126
s. translocations	6	8
s. inversions	0	0
Misassembled contigs	200	37
Misassembled contigs length	211889858	104831721
Local misassemblies	8122	2873
Scaffold gap ext. mis.	71	21
Scaffold gap loc. mis.	4856	1792
Unaligned mis. contigs	1502	563
Mismatches	412290	191921
Indels	95351	46834
Indels ($\le$ 5 bp)	67654	33342
Indels (> 5 bp)	27697	13492
Indels length	896205	436349

To investigate the refinement of the donkey genome in detail, we performed synteny comparison to the horse genome as shown in Fig. 3c, d using cutoffs of 0.1 and 0.2. The full alignment results were shown in Additional file 1: Figs. S23–S54. We first sorted donkey scaffolds and oriented them according to horse chromosomes (hereafter, labeled as ECA). Consider the absence of genomes from closest phylogenetic relatives(rhinos), we only take rearrangements occurring within donkey scaffolds as reliable. More strictly, only rearrangements that pass all cutoff thresholds are further investigated. We identified two inversions between the horse and the donkey, that mapped to ECA7 and ECA28, as shown in Fig. 4. Both of them have been verified in a de novo donkey genome by Chicago HiRise assembly [43]. Around regions these found inversions, LD was ranked top in genome-wide level, which is consistent with the algorithm that *LDscaff* used. Setting high cutoff can distinguish reliable inversions from false-positive ones caused by misassembly. It implies that *LDscaff* has the potential to aid in the identification of chromosome inversions.

### Comparison of HiC and *LDscaff*

We used HiC data to reassemble the same draft donkey genome from SOAPdenovo2, and the re-assembled scaffolds using LDscaff. The assembly was scaffolded with Hi-C data using the 3D-DNA pipeline [44]. The Hi-C reads were aligned to the draft donkey genome assembly using the Juicer pipeline [45]. The 3D-DNA pipeline was run with the default parameters. We evaluated assembly across four categories of error: relocations, translocation, inversion, and indels. The full comparison results are shown in Table 6. The number and size of these errors were calculated after QUAST splits input assembly by continuous fragments. For assembles involved HiC scaffolding, contigs shorter than 15 KB were set aside. Compared with HiC scaffolding, LDscaff-reassembled sequence has less scaffolding errors decreased with decreasing assembly contiguity. The His-reassembled sequence has twice misassembled bases more than the LDscaff result. When integrated HiC and LDscaff, the assembly size has decreased to 1.8G from 2.2G, while the N50 size has increased to 1.2G, which is the size of the longest scaffold.

**Table 4:** Comparison of the refined donkey genome with SOAPdenovo-assembled genome

Cutoff	Scaffolds before	Scaffolds after	N50 before	N50 after
0.1	80	81	23779253	2108947689
0.15	80	254	23779253	44040746
0.2	80	382	23779253	32092974
0.3	80	484	23779253	26289196
0.4	80	514	23779253	24644580
0.5	80	542	23779253	23779253

**Table 5:** Mis-assembly comparison of the refined donkey genome with SOAPdenovo-assembled genome

Assembly evaluation	Before LDscaff	After LDscaff
Misassemblies	7166	3266
Contig misassemblies	5173	2330
c. relocations	1517	651
c. translocations	3319	1522
c. inversions	337	157
Scaffold misassemblies	1993	936
s. relocations	425	215
s. translocations	1543	712
s. inversions	25	9
Misassembled contigs	369	122
Misassembled contigs length	2256451641	1112560100
Local misassemblies	59490	26941
Scaffold gap ext. mis.	80	31
Scaffold gap loc. mis.	26321	12206
Unaligned mis. contigs	18	26
Mismatches	27642582	13299554
Indels	2673003	1283993
Indels ($\le$ 5 bp)	2189565	1051168
Indels (> 5 bp)	483438	232825
Indels length	13686335	6581365

## Discussion

Assemble the sequenced reads to chromosome level is a long term puzzle in genome analysis. The Human Genome Project tool scientists about 10 years to complete that first human genome sequence. Currently, tools are capable of obtaining a more accurate human genome with only hundreds of CPU hours [46].

With the explosive volume of sequencing data, it is important to take full advantage of the features in different sequencing strategies to achieve the task of genome assembly. Short reads provide accurate base calls while long reads can help to reconstruct the long-range structure of the genome [47]. Linked-Reads group reads deriving from the same molecule [48]. Hi-C data can provide linkage information across a variety of length scales. LD from population SNP data offers patterns of recombination rates. Hybrid assembly tools have been developed for integrating all these sequencing techniques [49]. Most of them are based on a clustering-assembly strategy by solving the scaffold orientation and order asynchronously. It has been approved that the hybrid strategy can boost the assembly result in both continuity and accuracy.

LD indicates the non-random associations between physical markers. Linkage information among genes or loci provides relative position information [23] and is capable of finding scaffolds that conflict with their relative orders. Genetic linkage maps have been used to refine the de novo genome assemblies [6, 50], and show the potential to guide the layout of scaffolds. However, with millions of genomes are now being collected, the information provided by population data has not been fully utilized to resolve the task of genome assembly. Here we proposed a computational graph-based algorithm to resolve the scaffold orders and orientation simultaneously. Our method proves to be effective when applied to both simulated data and empirical data.

The sample size affects LD estimation. Theoretically, more individuals sampled leads to better performance. Two main methods are used to calculate LD, $r^{2}$ and $\left| {D}' \right|$. $r^{2}$ has been proved not noticeably affected by sample size [51], which *LDscaff* uses. A minimum sample size of 55 for accurate calculation of LD is suggested [51].

Linkage information can help increase the continuity of assembly, but locus pairs with too long distances between them provide weak linkage power. Thereby we cut the unreliable links with a proper cutoff threshold. We advance the cutoff threshold should be equal to or larger than 0.2. The larger the cutoff threshold, the less the assembled scaffold contiguity, but better accuracy.

**Table 6:** Assembly comparison after spliting by continuous fragments

Assembly composition	SOAPdenovo + LDscaff	SOAPdenovo + HiC	SOAPdenovo + LDscaff + HiC
Assembly size	2266537396	2252886107	1797011536
No. scaffolds	382	647	123
N50 size	32092974	58665385	1254064180
Misassemblies	2426	4852	2162
Contig misassemblies	2426	4852	2162
c. relocations	589	1231	524
c. translocations	1678	3298	1490
c. inversions	159	323	148
Scaffold misassemblies	0	0	0
s. relocations	0	0	0
s. translocations	0	0	0
s. inversions	0	0	0
Misassembled contigs	1528	3120	1384
Misassembled contigs length	71602181	144952053	67084793
Local misassemblies	28366	58360	27273
Unaligned mis. contigs	117	203	102
Mismatches	13257465	27321894	12732835
Indels	1249703	2585745	1204396
Indels ($\le$ 5 bp)	1041691	2156454	1004988
Indels (> 5 bp)	208012	429291	199408
Indels length	5760596	11919093	5519229

## Conclusions

We hope that the improvement in genome assembly provided by *LDscaff* will further boost the use of existed sequencing data. While assemblers improve genome assemblies, data they require is the cost to be considered. Therefore, *LDscaff* provides an example to improve assembly quality by mining biological databases.

## Supplementary information

**Additional file 1**. The dot plots of LD-scaff re-assemblied scaffolds in Panda and Donkey genome.

## Data Availability

The swine WGS data were collected from the Pig variations and Positive Selection (PigVar) database, the swine assembly (Sus scrofa 10.2) can be obtained under GCA_000003025.4. We downloaded the giant panda reference (AilMel 1.0) from the NCBI GenBank database (Accession number: GCA000004335.1). We downloaded the panda GWHACDL00000000 assembly from National Genomics Data Center. The panda WGS data can be obtained under accession SRA053353. The data sets of donkey individuals generated and analyzed during the current study are not publicly available due to these data have been cited from another paper that is being peer-reviewed. Data sharing will be applied later. Project name: LDscaff; Project home page: https://github.com/YingXiaoZhou/LDscaff; Operating system(s): Platform independent; Programming language: Shell script, c++; Other requirements: lemon library installed; License: see web page; Any restrictions to use by non-academics: licence needed.

## Abbreviations

NGS: Next Generation Sequencing
LD: Linkage disequilibrium
WGS: Whole genome sequencing
SRA: Short Read Archive
PigVar: Pig variations and Positive Selection
