# Supplementary material for: LDscaff: LD-based scaffolding of de novo genome assemblies
Source: BMC Bioinformatics. 2020 Dec 28;21(Suppl 21):570. doi: 10.1186/s12859-020-03895-7 (PMC7768660; doi:10.1186/s12859-020-03895-7)
Supplement: Supplementary file 1 — Additional file 1. The dot plots of LD-scaff re-assemblied scaffolds in Panda and Donkey genome. [file 12859_2020_3895_MOESM1_ESM.pdf]

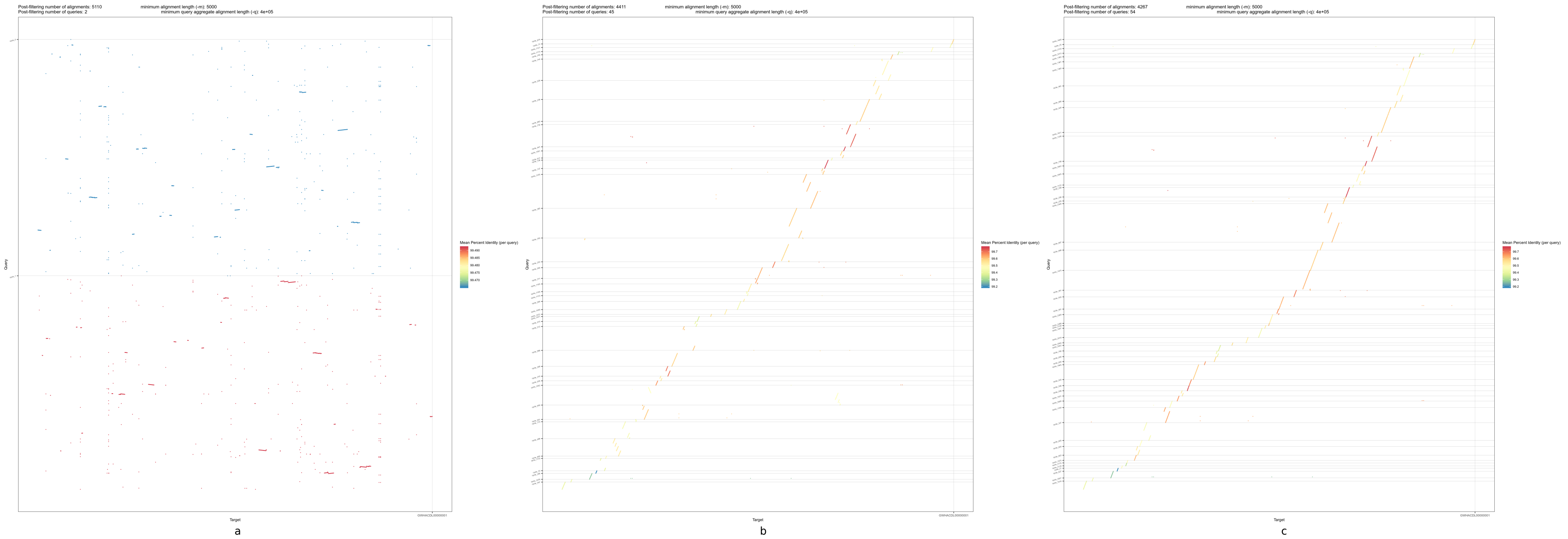

Figure S1: The dot plots of re-assembled scaffolds and the Panda reference chromosome 1 using different cutoffs. (a) cutoff=0.1; (b) cutoff=0.2; (c)cutoff=0.3.

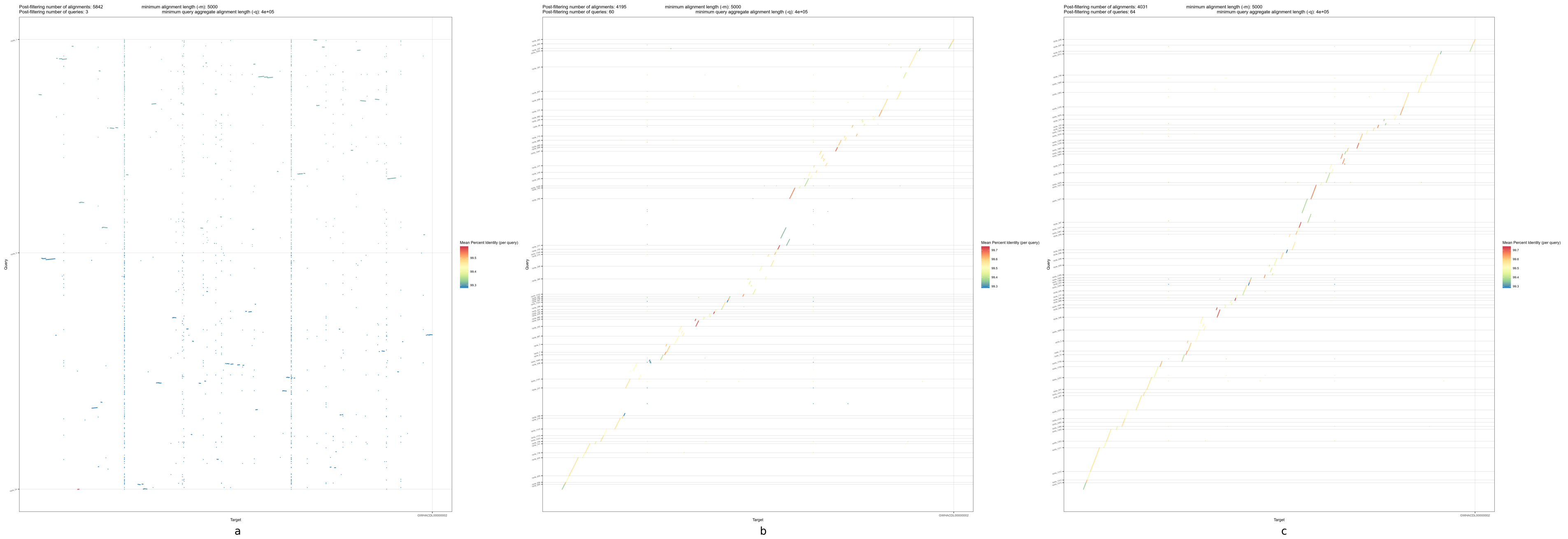

Figure S2: The dot plots of re-assembled scaffolds and the Panda reference chromosome 2 using different cutoffs. (a) cutoff=0.1; (b) cutoff=0.2; (c)cutoff=0.3.

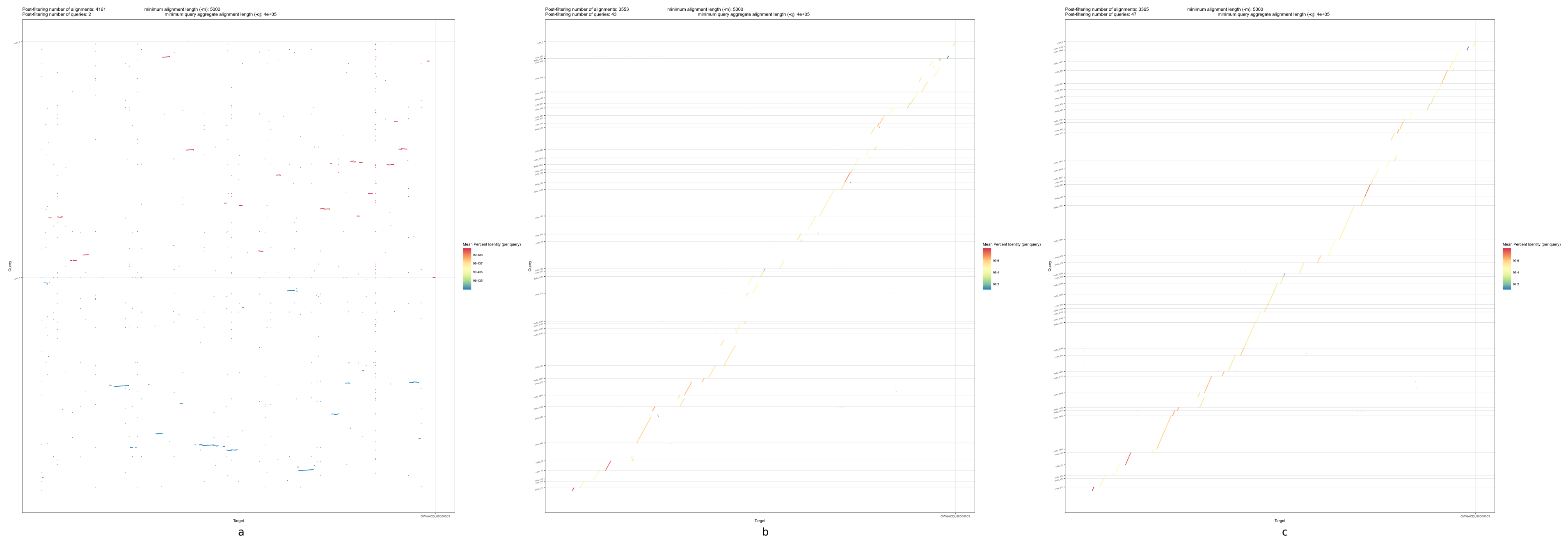

Figure S3: The dot plots of re-assembled scaffolds and the Panda reference chromosome 3 using different cutoffs. (a) cutoff=0.1; (b) cutoff=0.2; (c)cutoff=0.3.

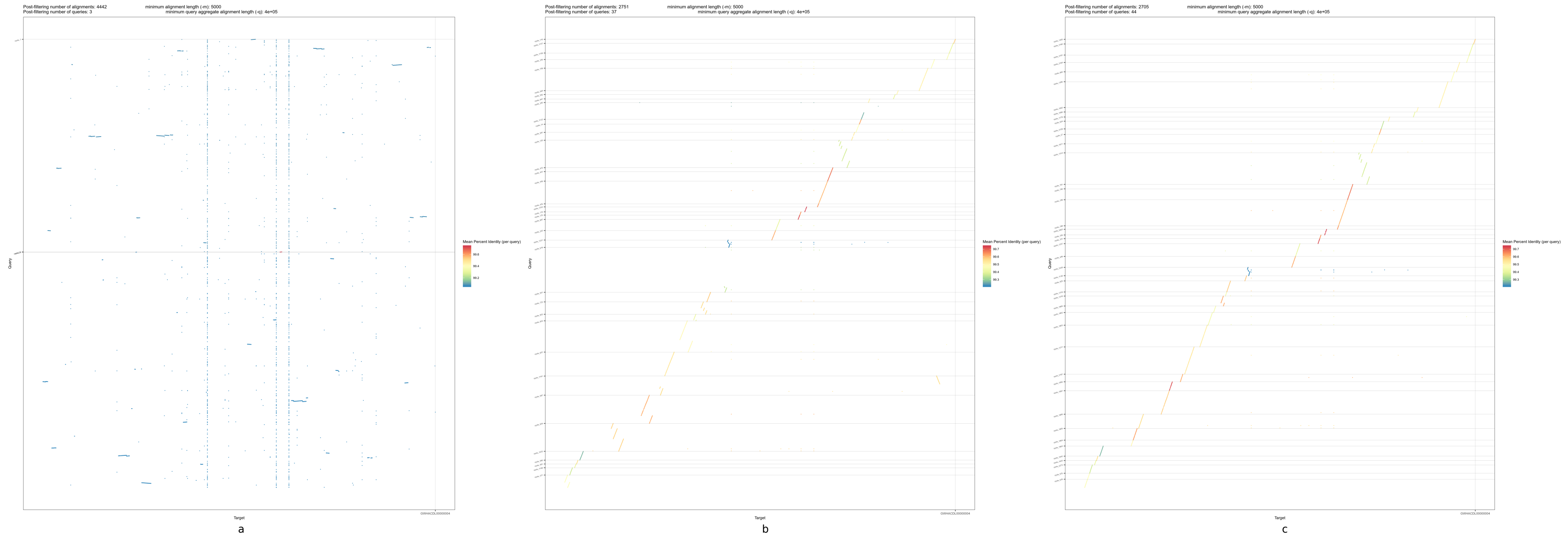

Figure S4: The dot plots of re-assembled scaffolds and the Panda reference chromosome 4 using different cutoffs. (a) cutoff=0.1; (b) cutoff=0.2; (c)cutoff=0.3.

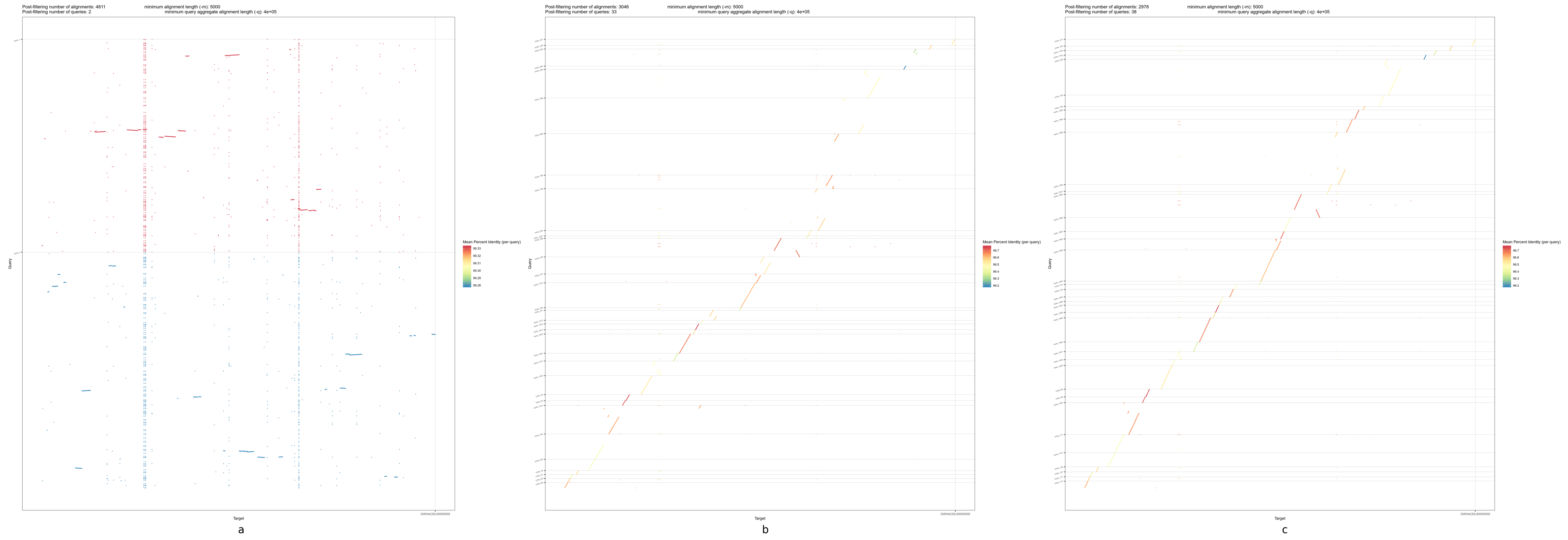

Figure S5: The dot plots of re-assembled scaffolds and the Panda reference chromosome 5 using different cutoffs. (a) cutoff=0.1; (b) cutoff=0.2; (c)cutoff=0.3.

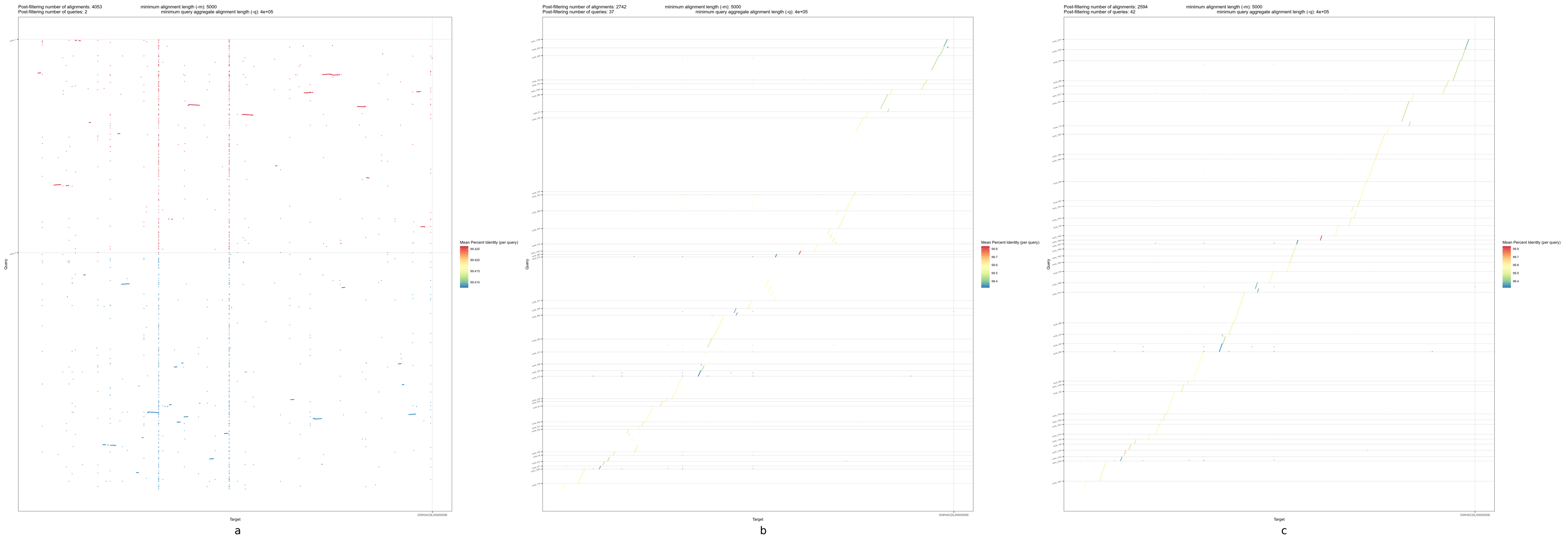

Figure S6: The dot plots of re-assembled scaffolds and the Panda reference chromosome 6 using different cutoffs. (a) cutoff=0.1; (b) cutoff=0.2; (c)cutoff=0.3.

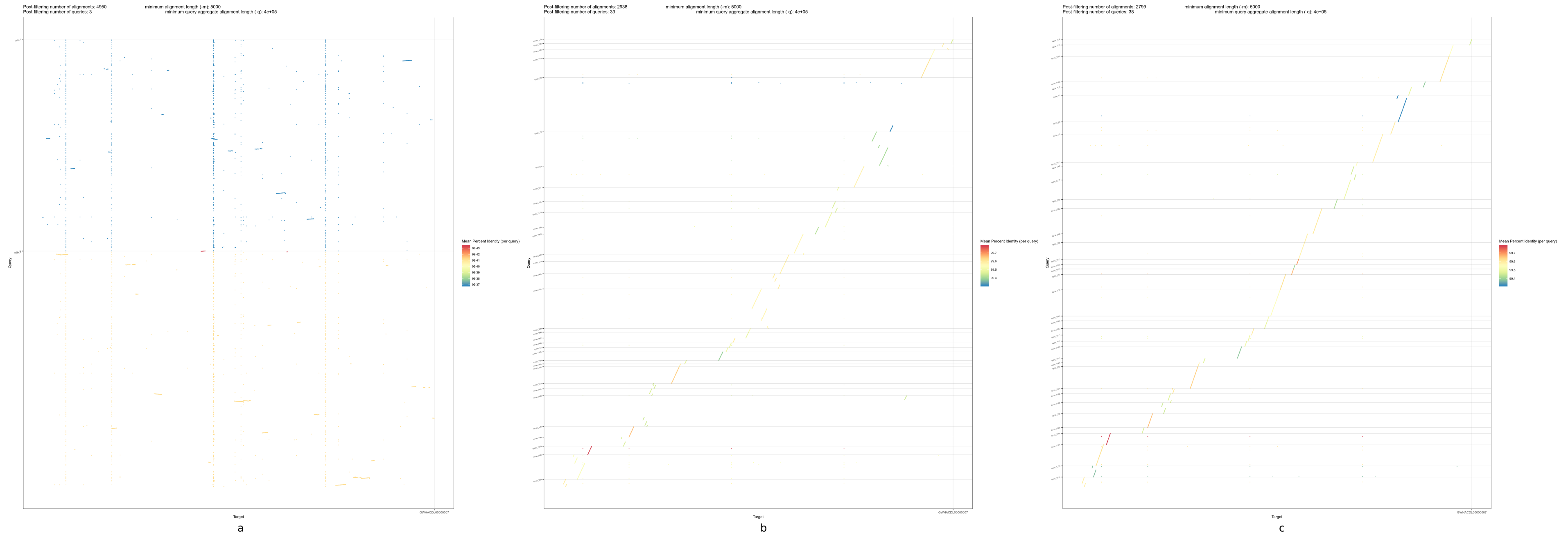

Figure S7: The dot plots of re-assembled scaffolds and the Panda reference chromosome 7 using different cutoffs. (a) cutoff=0.1; (b) cutoff=0.2; (c)cutoff=0.3.

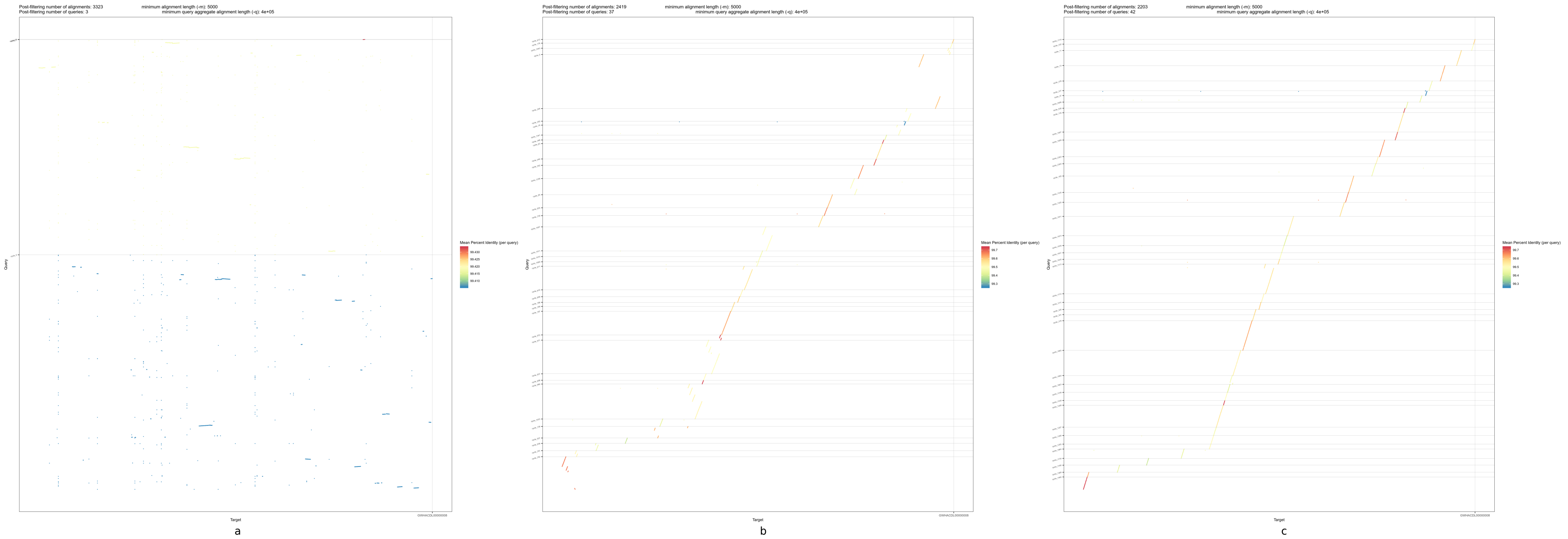

Figure S8: The dot plots of re-assembled scaffolds and the Panda reference chromosome 8 using different cutoffs. (a) cutoff=0.1; (b) cutoff=0.2; (c)cutoff=0.3.

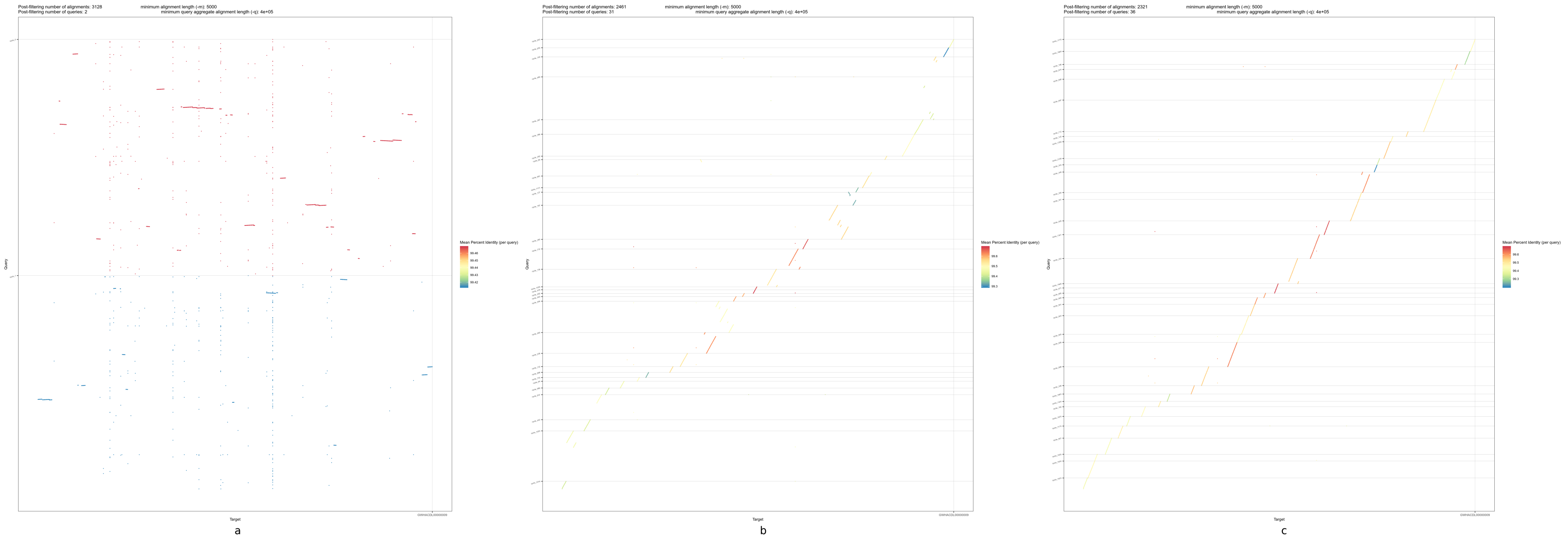

Figure S9: The dot plots of re-assembled scaffolds and the Panda reference chromosome 9 using different cutoffs. (a) cutoff=0.1; (b) cutoff=0.2; (c)cutoff=0.3.

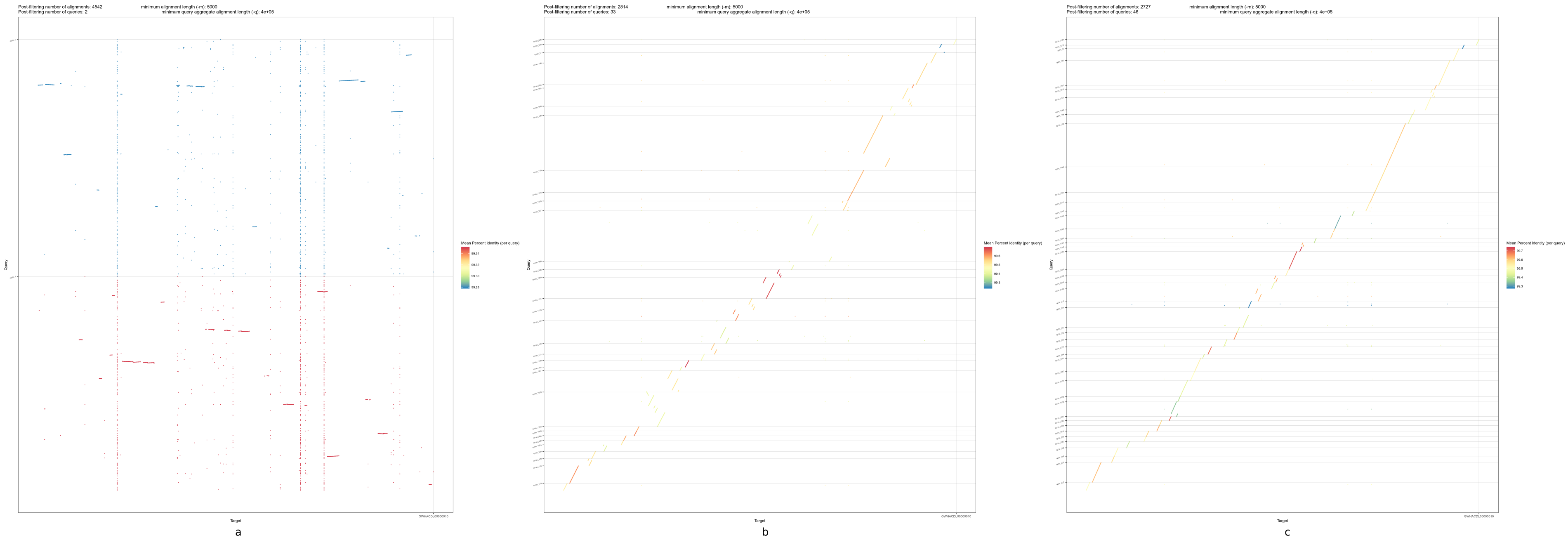

Figure S10: The dot plots of re-assembled scaffolds and the Panda reference chromosome 10 using different cutoffs. (a) cutoff=0.1; (b) cutoff=0.2; (c)cutoff=0.3.

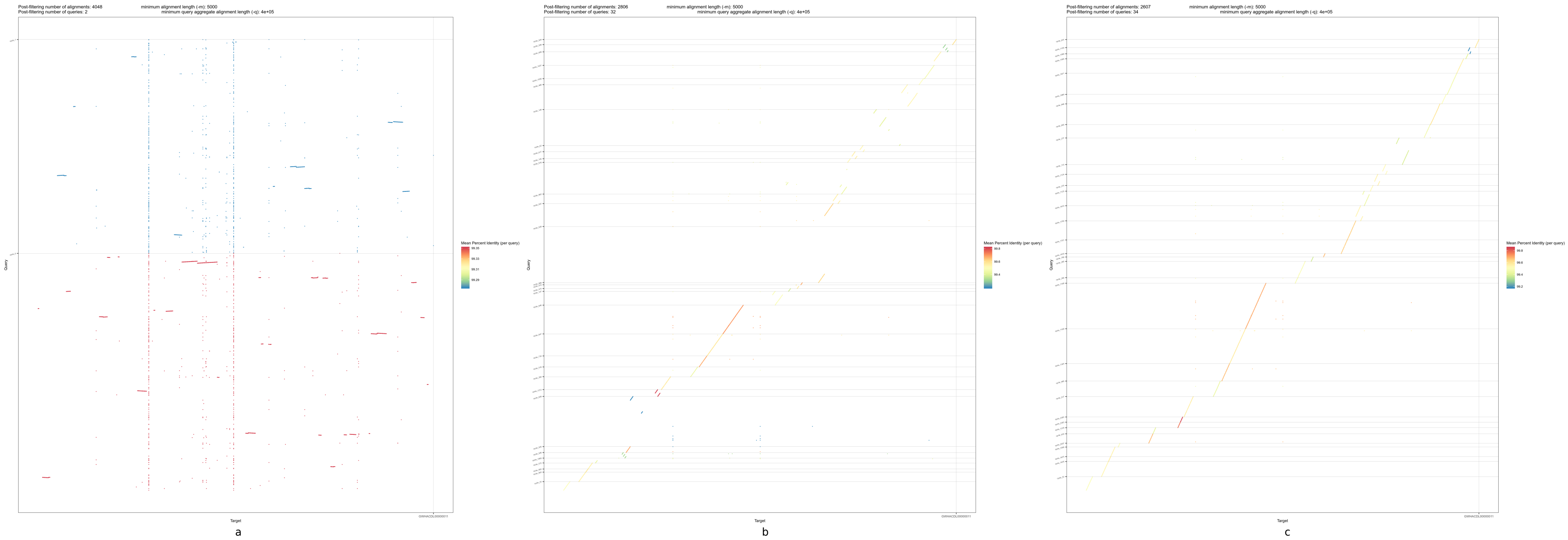

Figure S11: The dot plots of re-assembled scaffolds and the Panda reference chromosome 11 using different cutoffs. (a) cutoff=0.1; (b) cutoff=0.2; (c)cutoff=0.3.

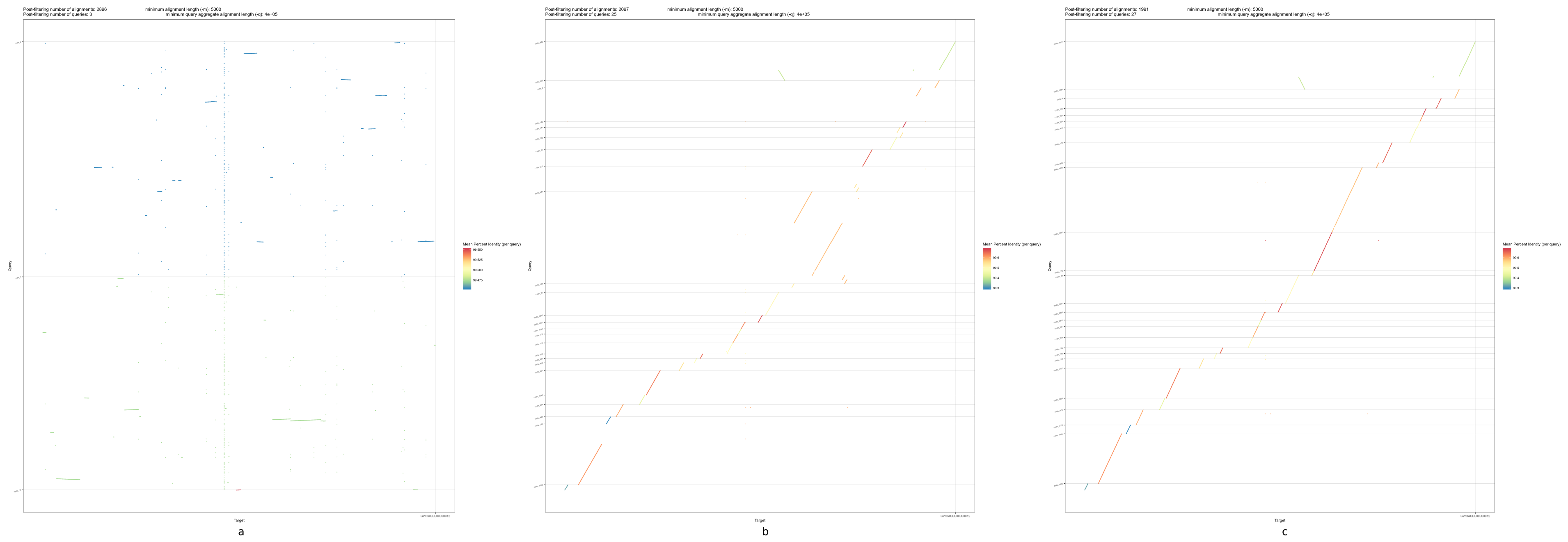

Figure S12: The dot plots of re-assembled scaffolds and the Panda reference chromosome 12 using different cutoffs. (a) cutoff=0.1; (b) cutoff=0.2; (c)cutoff=0.3.

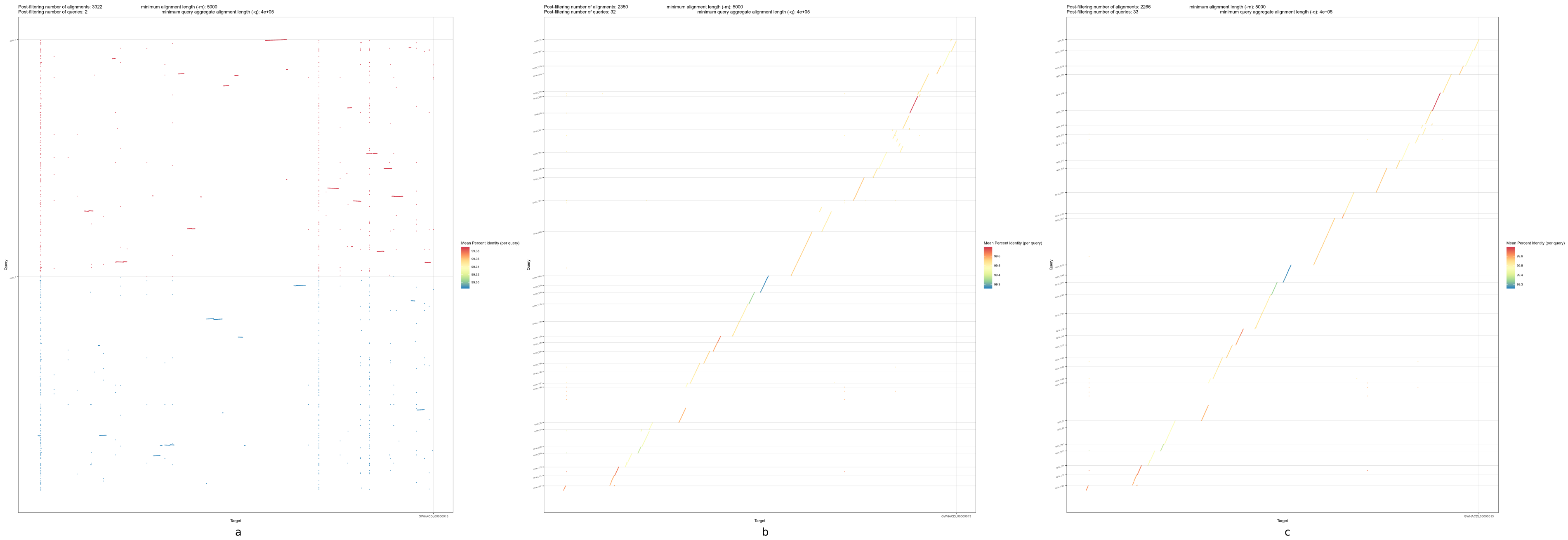

Figure S13: The dot plots of re-assembled scaffolds and the Panda reference chromosome 13 using different cutoffs. (a) cutoff=0.1; (b) cutoff=0.2; (c)cutoff=0.3.

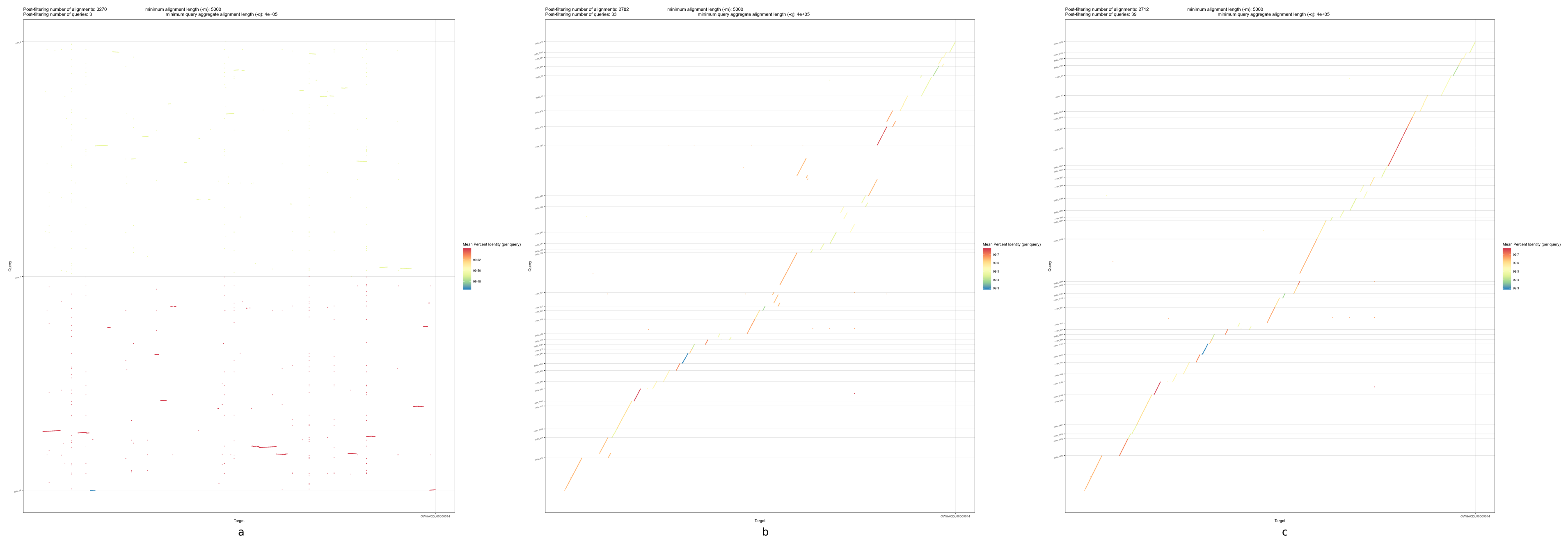

Figure S14: The dot plots of re-assembled scaffolds and the Panda reference chromosome 14 using different cutoffs. (a) cutoff=0.1; (b) cutoff=0.2; (c)cutoff=0.3.

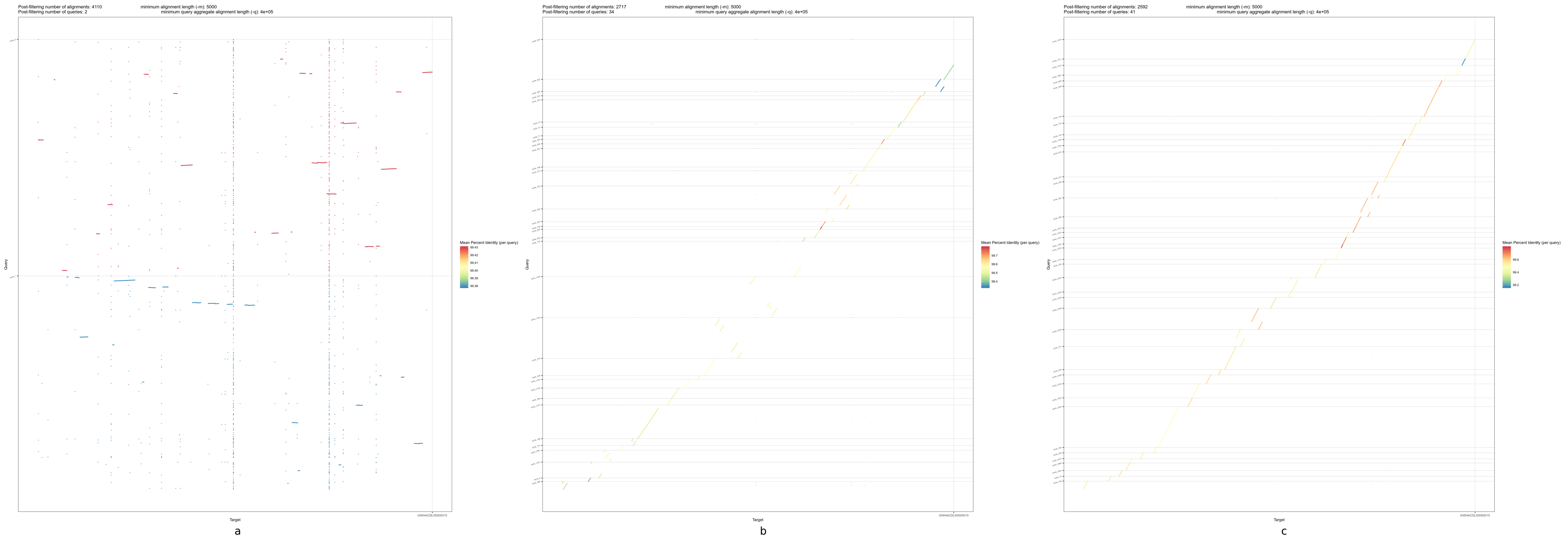

Figure S15: The dot plots of re-assembled scaffolds and the Panda reference chromosome 15 using different cutoffs. (a) cutoff=0.1; (b) cutoff=0.2; (c)cutoff=0.3.

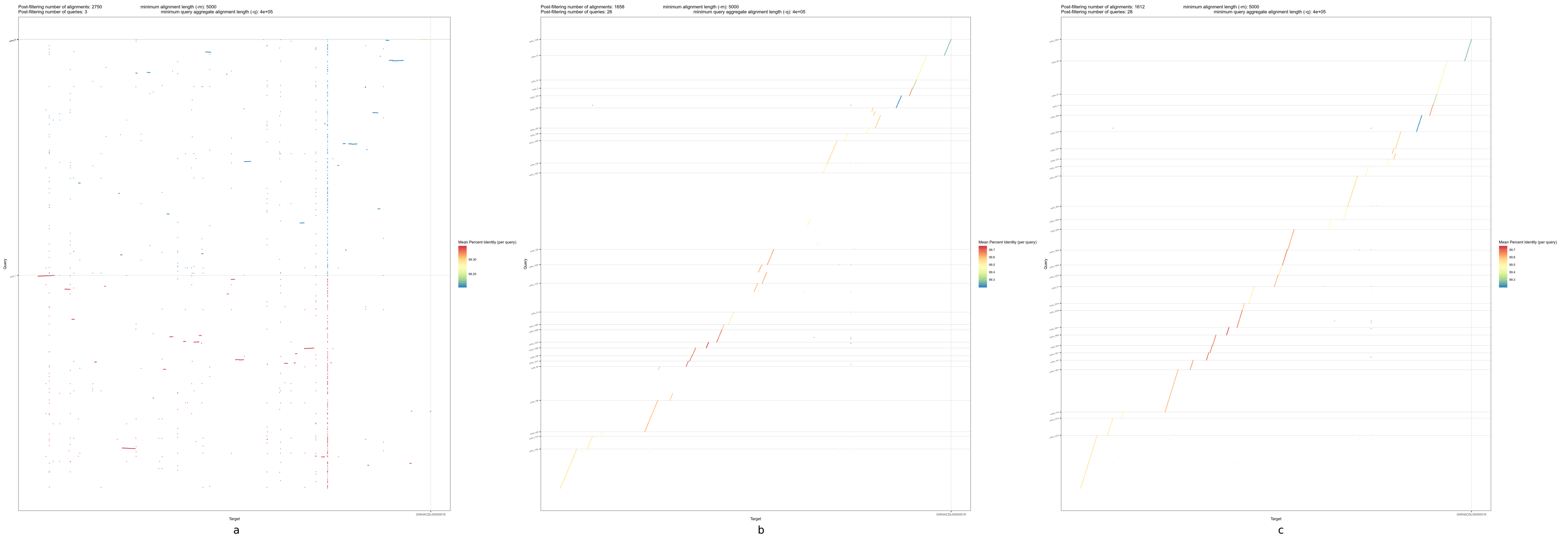

Figure S16: The dot plots of re-assembled scaffolds and the Panda reference chromosome 16 using different cutoffs. (a) cutoff=0.1; (b) cutoff=0.2; (c)cutoff=0.3.

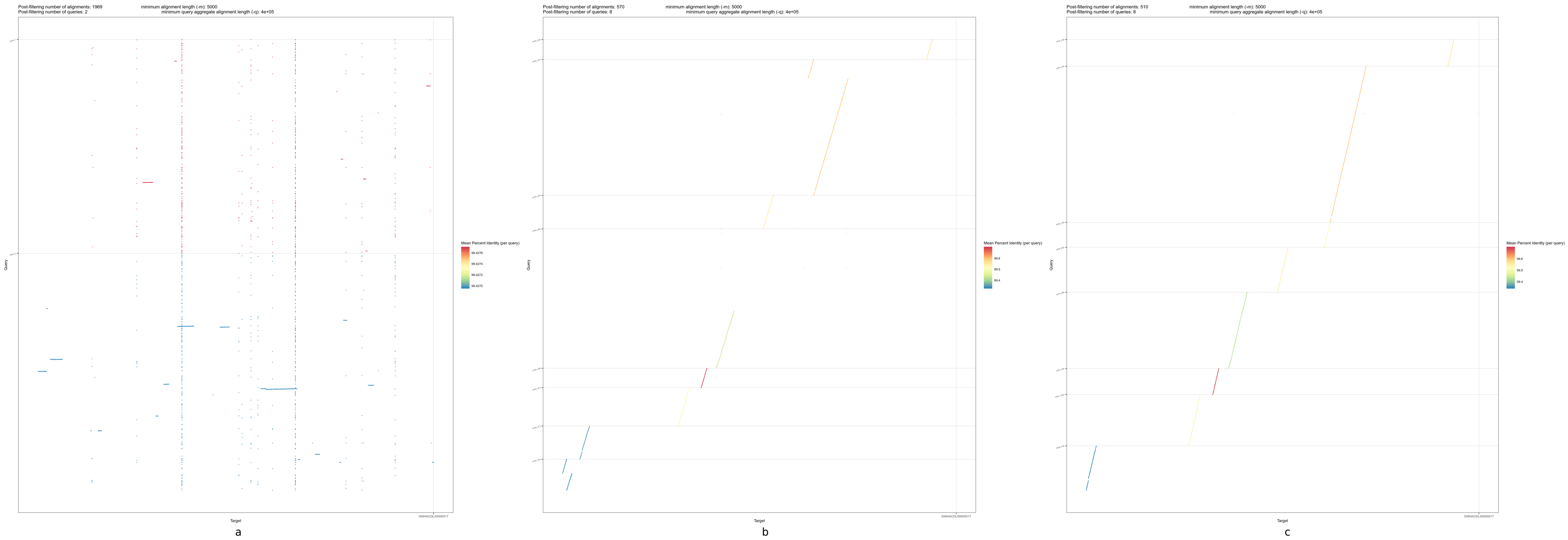

Figure S17: The dot plots of re-assembled scaffolds and the Panda reference chromosome 17 using different cutoffs. (a) cutoff=0.1; (b) cutoff=0.2; (c)cutoff=0.3.

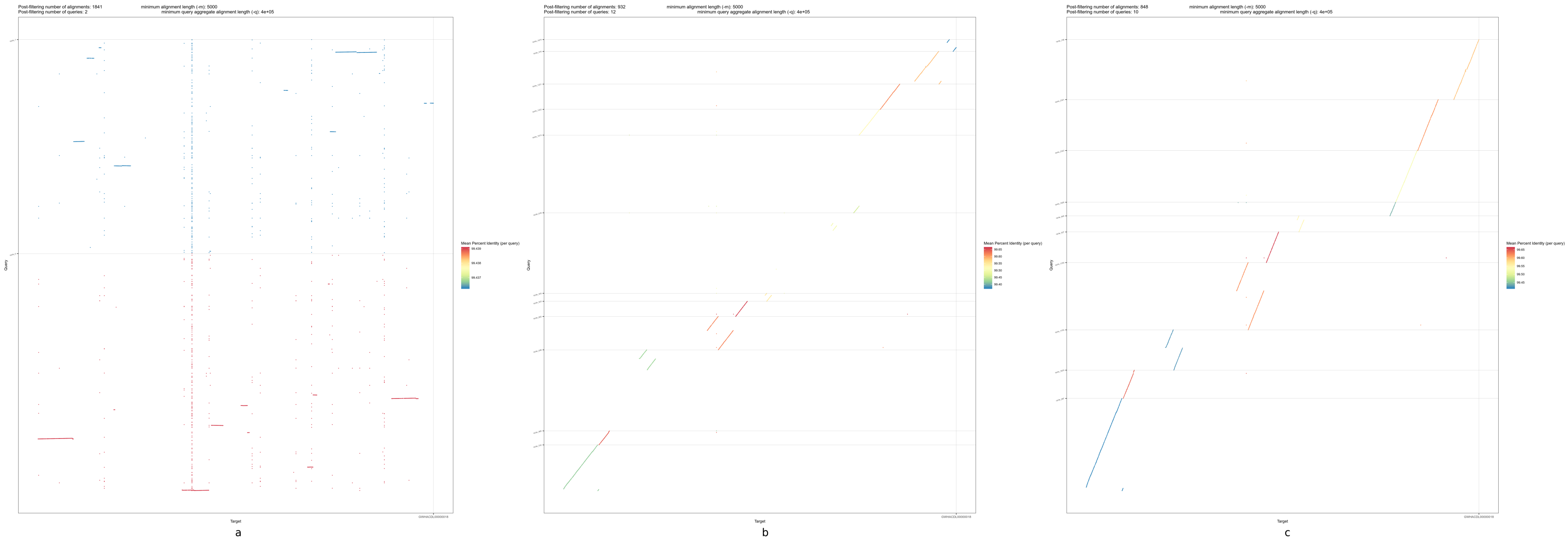

Figure S18: The dot plots of re-assembled scaffolds and the Panda reference chromosome 18 using different cutoffs. (a) cutoff=0.1; (b) cutoff=0.2; (c)cutoff=0.3.

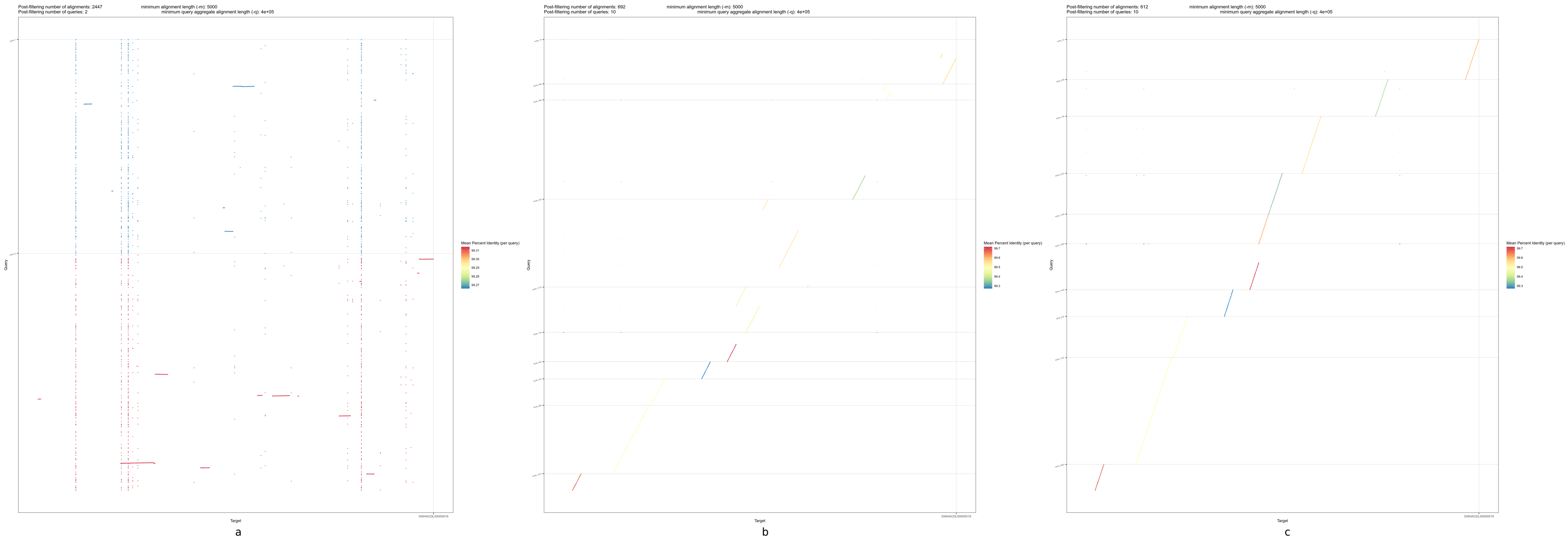

Figure S19: The dot plots of re-assembled scaffolds and the Panda reference chromosome 19 using different cutoffs. (a) cutoff=0.1; (b) cutoff=0.2; (c)cutoff=0.3.

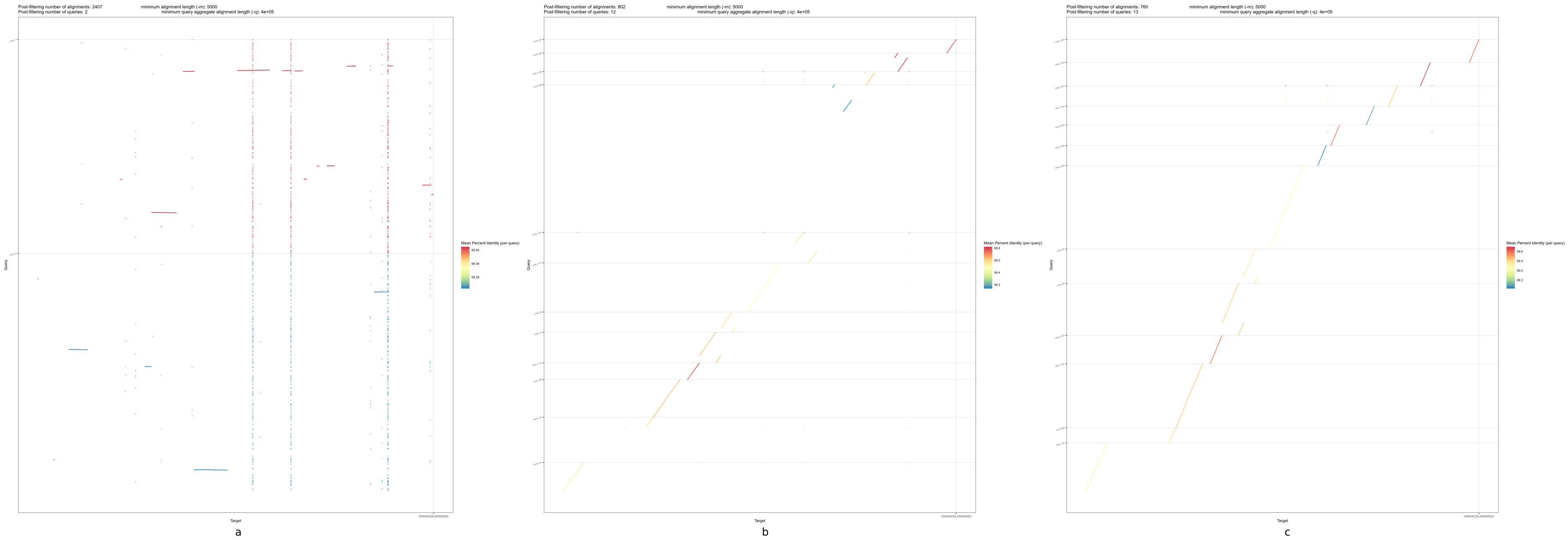

Figure S20: The dot plots of re-assembled scaffolds and the Panda reference chromosome 20 using different cutoffs. (a) cutoff=0.1; (b) cutoff=0.2; (c)cutoff=0.3.

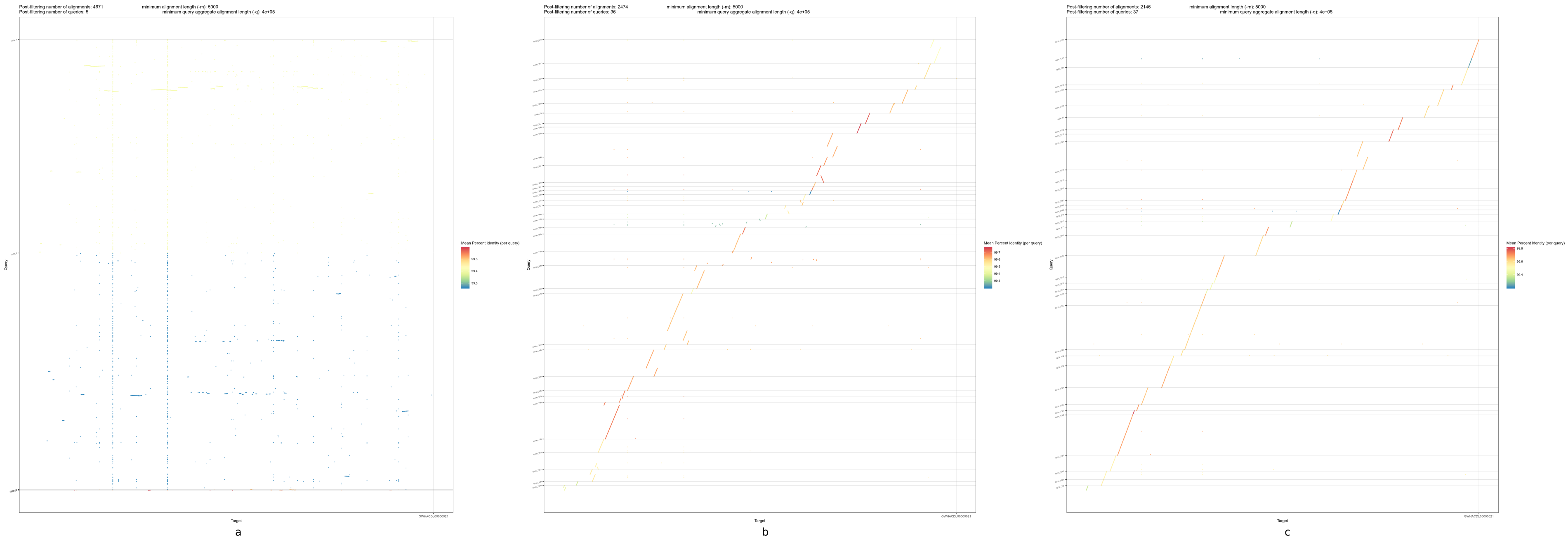

Figure S21: The dot plots of re-assembled scaffolds and the Panda reference chromosome 21 using different cutoffs. (a) cutoff=0.1; (b) cutoff=0.2; (c)cutoff=0.3.

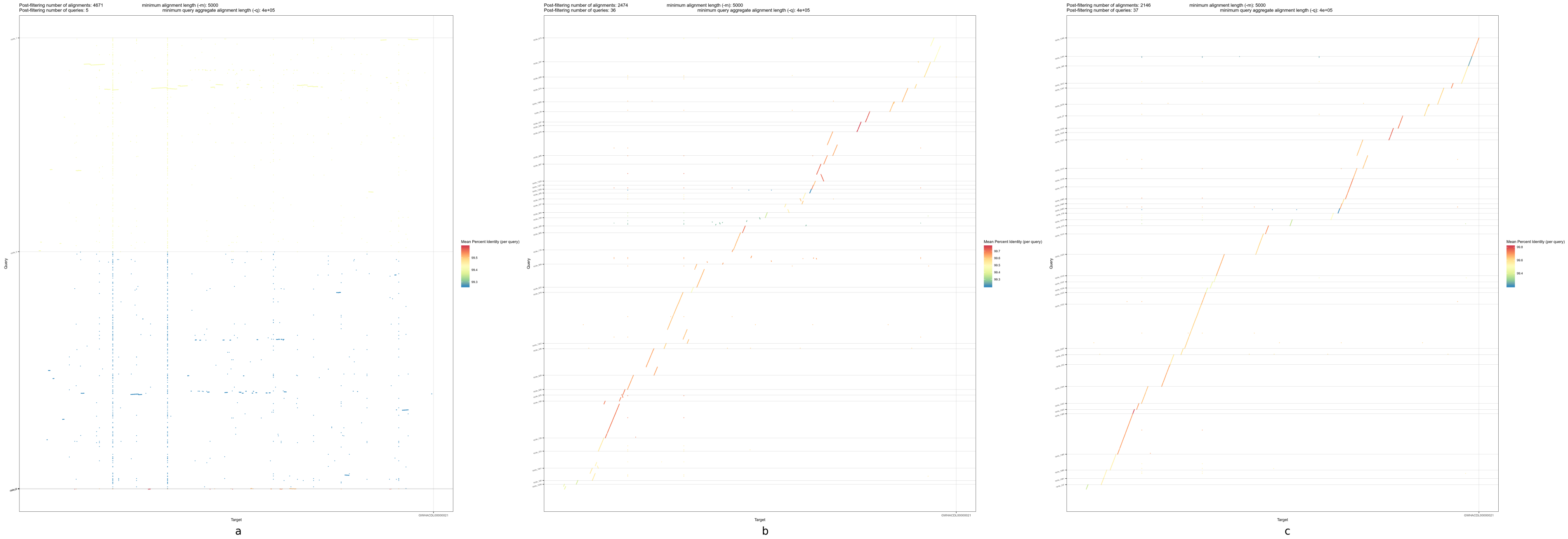

Figure S22: The dot plots of re-assembled scaffolds and the Panda reference chromosome X using different cutoffs. (a) cutoff=0.1; (b) cutoff=0.2; (c)cutoff=0.3.

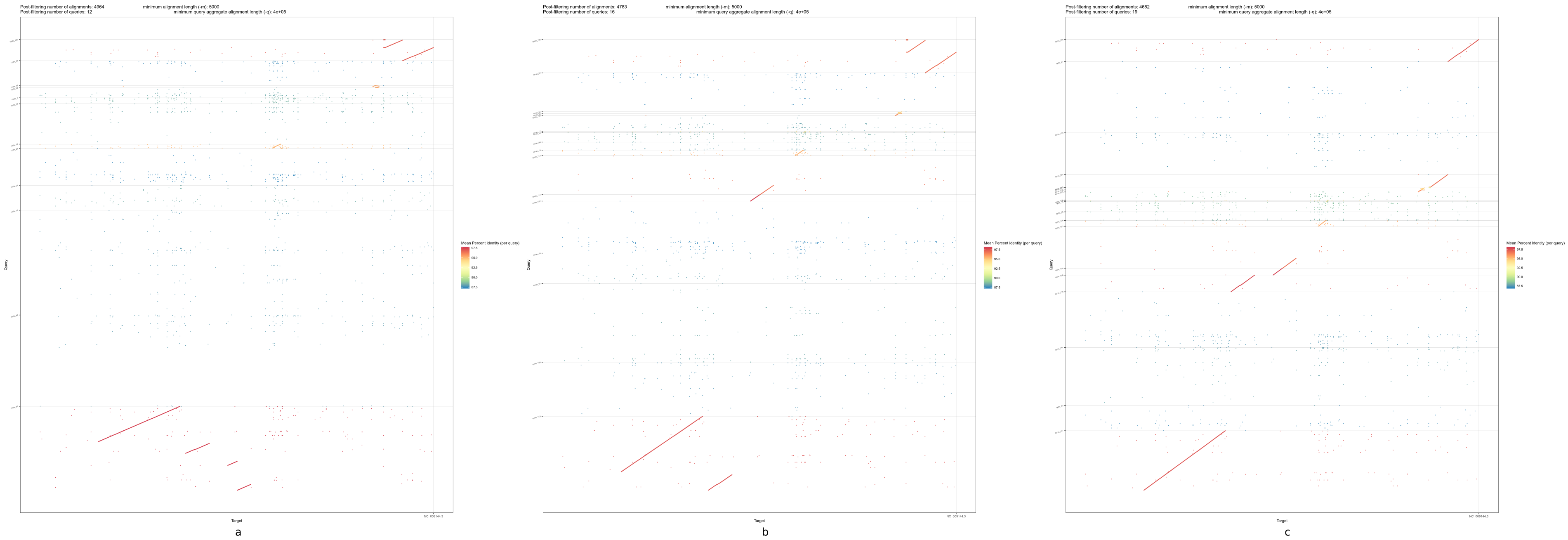

Figure S23: The dot plots of re-assembled scaffolds and the donkey reference chromosome 1 using different cutoffs. (a) cutoff=0.1; (b) cutoff=0.2; (c)cutoff=0.3.

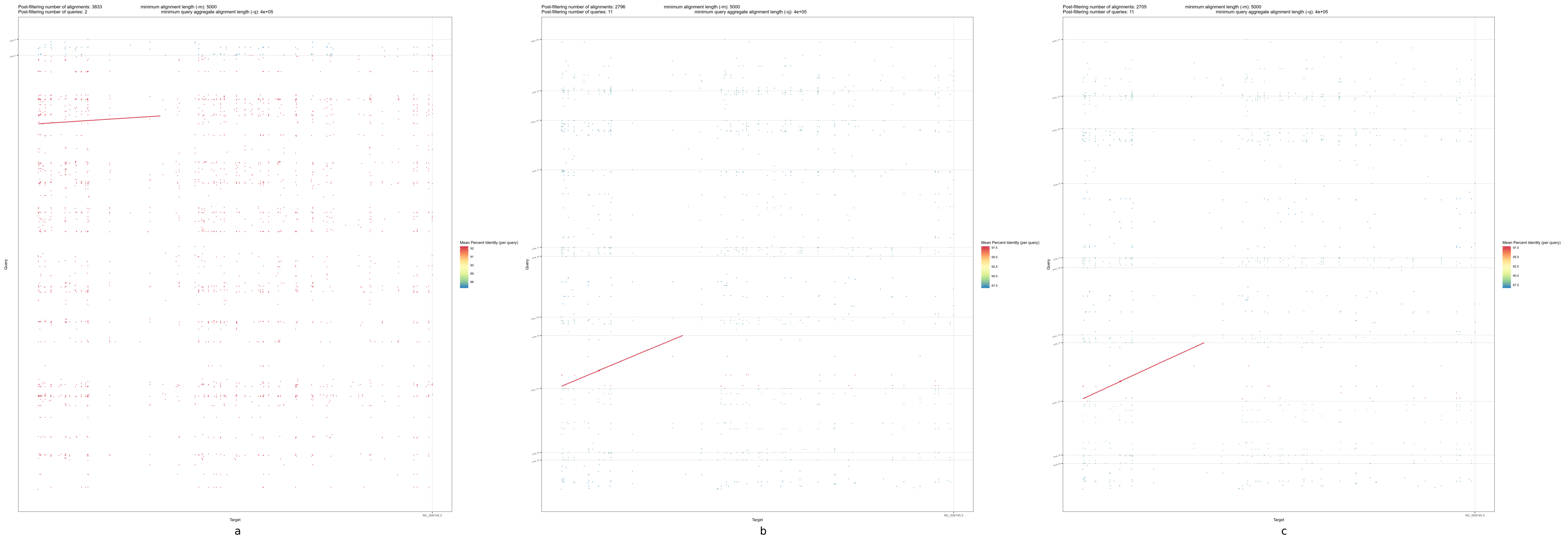

Figure S24: The dot plots of re-assembled scaffolds and the donkey reference chromosome 2 using different cutoffs. (a) cutoff=0.1; (b) cutoff=0.2; (c)cutoff=0.3.

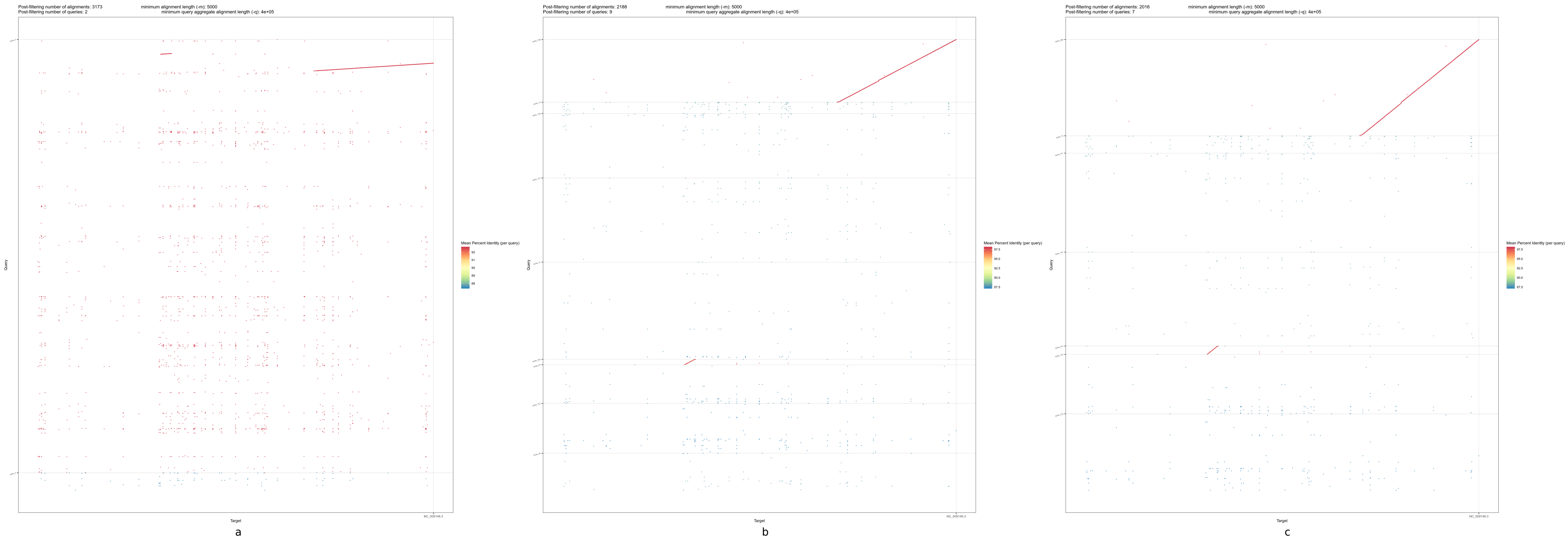

Figure S25: The dot plots of re-assembled scaffolds and the donkey reference chromosome 3 using different cutoffs. (a) cutoff=0.1; (b) cutoff=0.2; (c)cutoff=0.3.

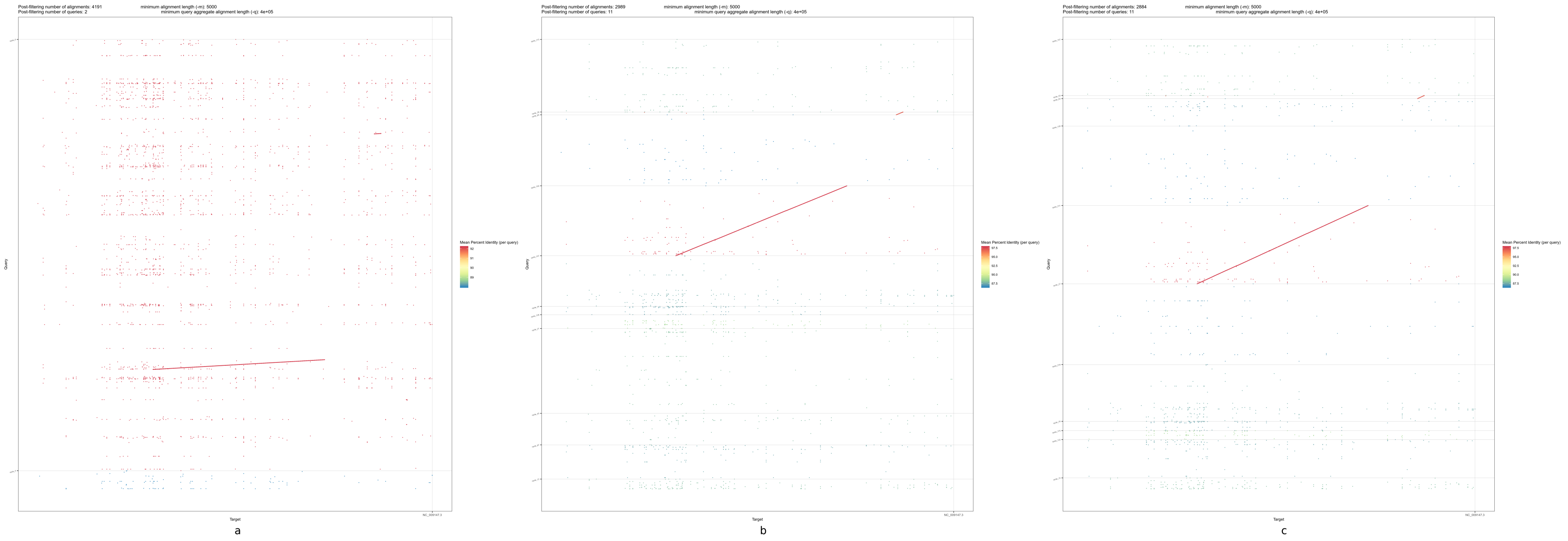

Figure S26: The dot plots of re-assembled scaffolds and the donkey reference chromosome 4 using different cutoffs. (a) cutoff=0.1; (b) cutoff=0.2; (c)cutoff=0.3.

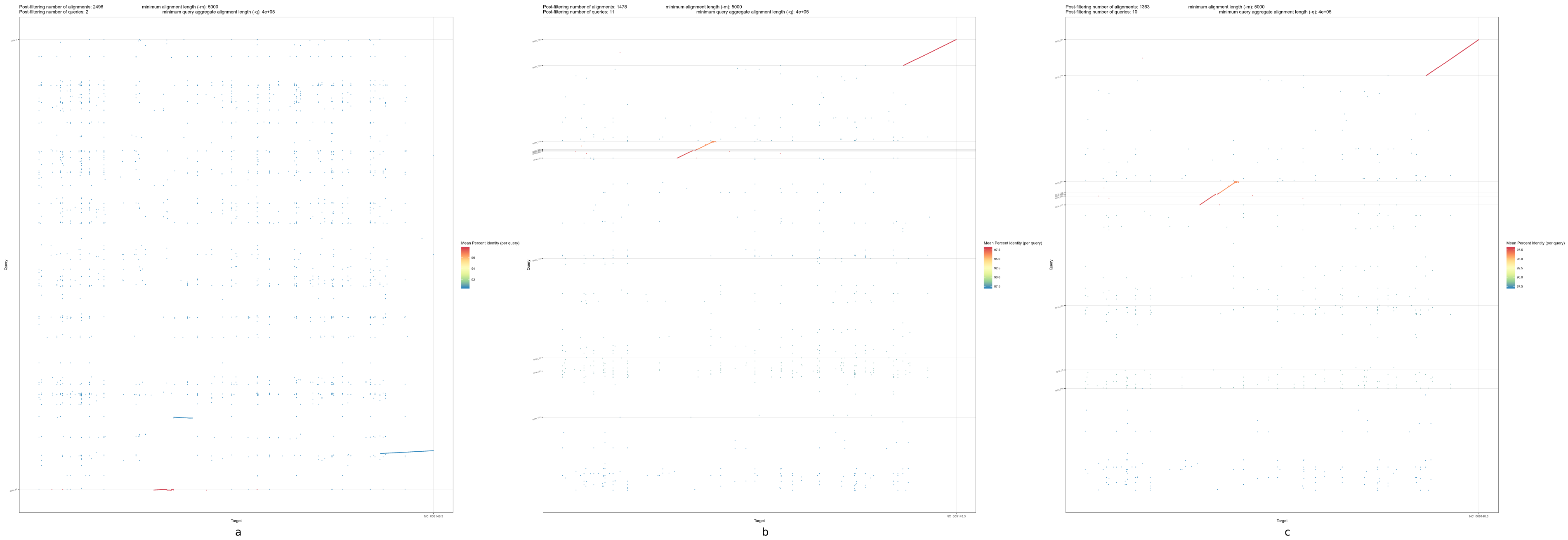

Figure S27: The dot plots of re-assembled scaffolds and the donkey reference chromosome 5 using different cutoffs. (a) cutoff=0.1; (b) cutoff=0.2; (c)cutoff=0.3.

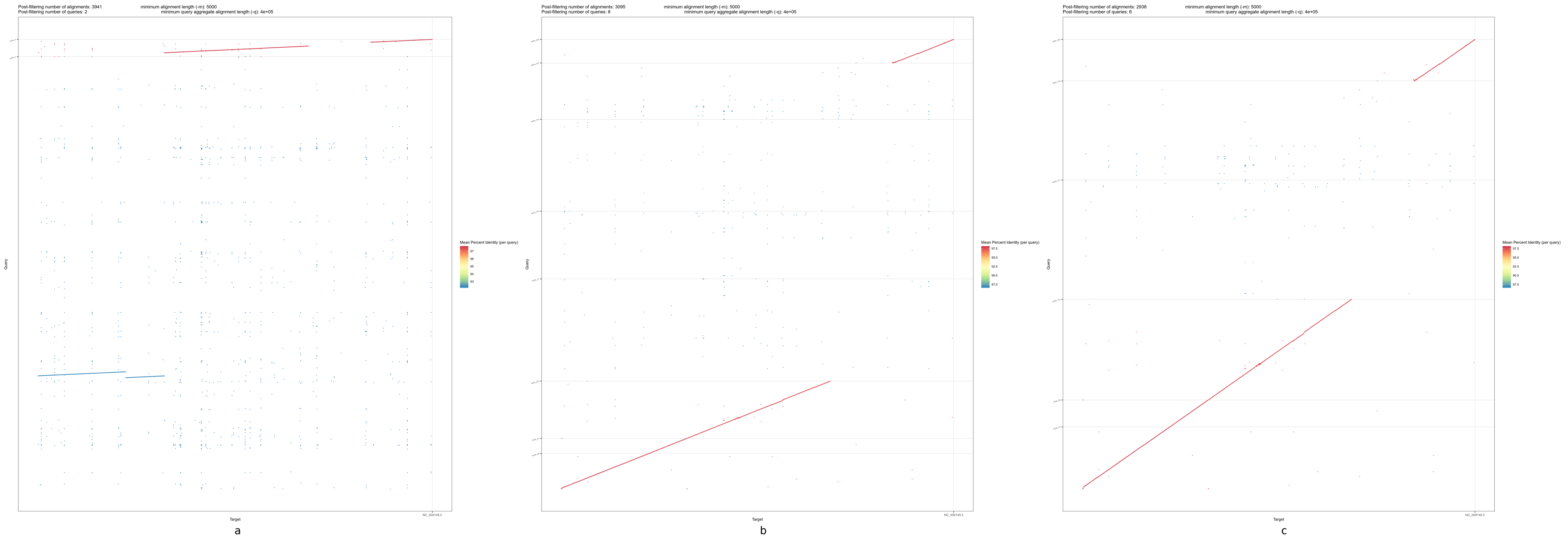

Figure S28: The dot plots of re-assembled scaffolds and the donkey reference chromosome 6 using different cutoffs. (a) cutoff=0.1; (b) cutoff=0.2; (c)cutoff=0.3.

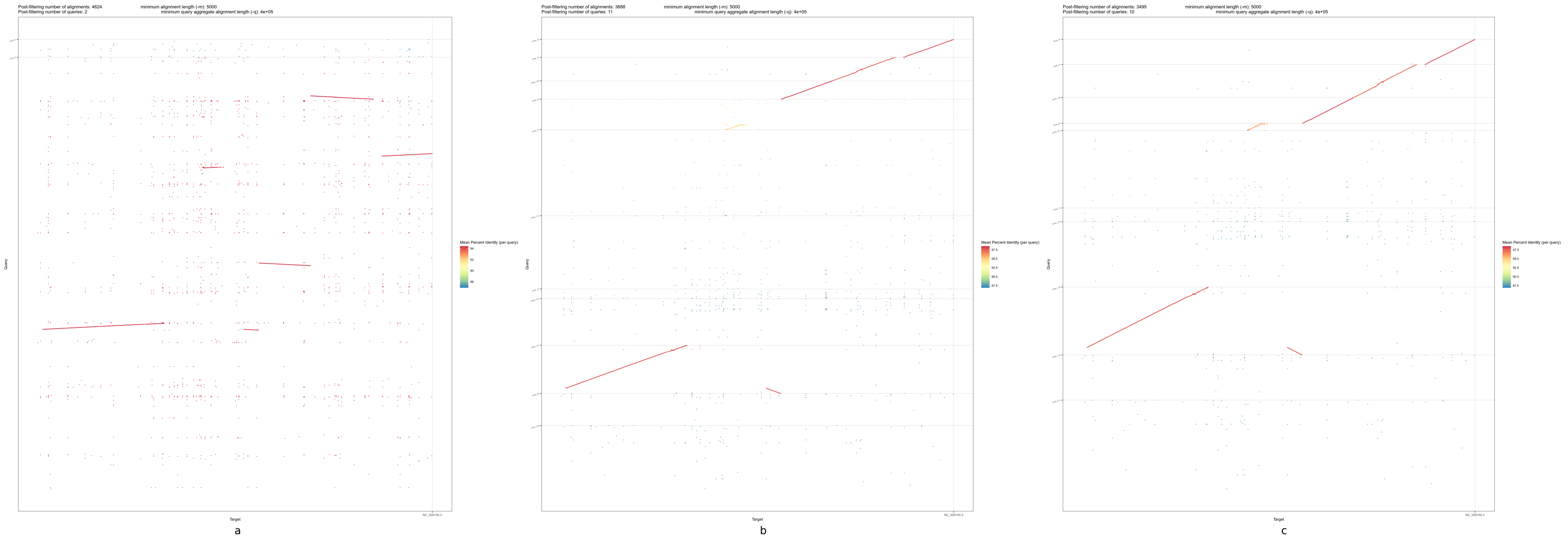

Figure S29: The dot plots of re-assembled scaffolds and the donkey reference chromosome 7 using different cutoffs. (a) cutoff=0.1; (b) cutoff=0.2; (c)cutoff=0.3.

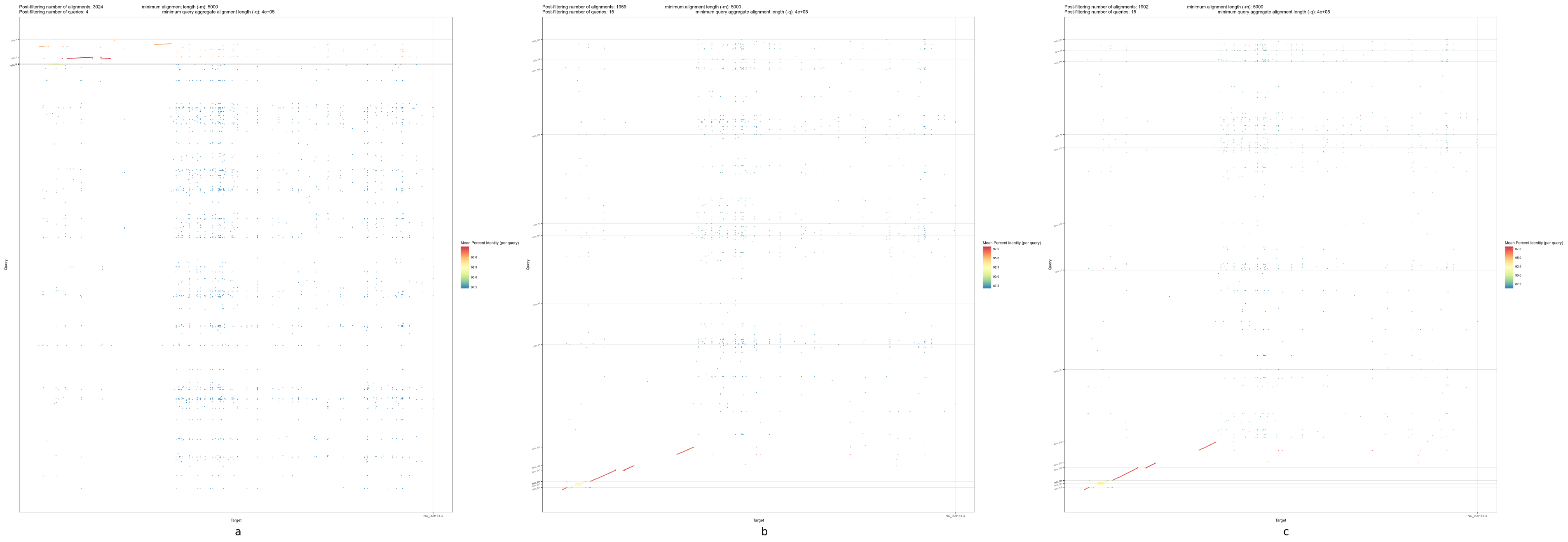

Figure S30: The dot plots of re-assembled scaffolds and the donkey reference chromosome 8 using different cutoffs. (a) cutoff=0.1; (b) cutoff=0.2; (c)cutoff=0.3.

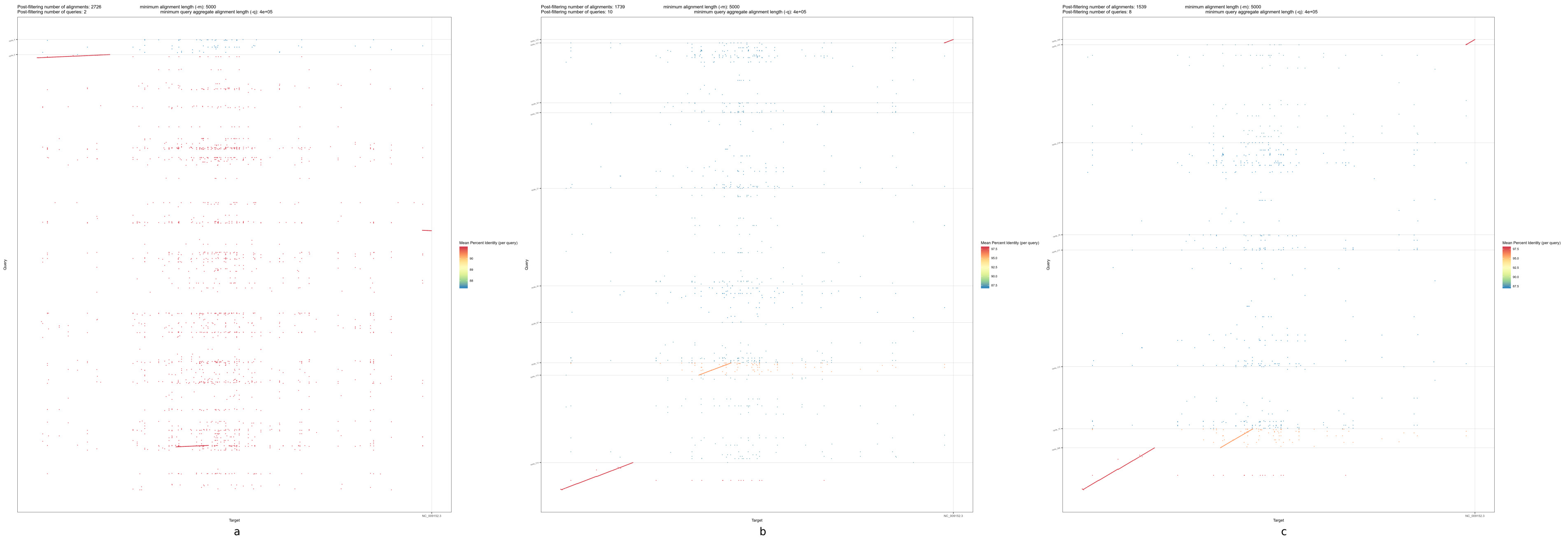

Figure S31: The dot plots of re-assembled scaffolds and the donkey reference chromosome 9 using different cutoffs. (a) cutoff=0.1; (b) cutoff=0.2; (c)cutoff=0.3.

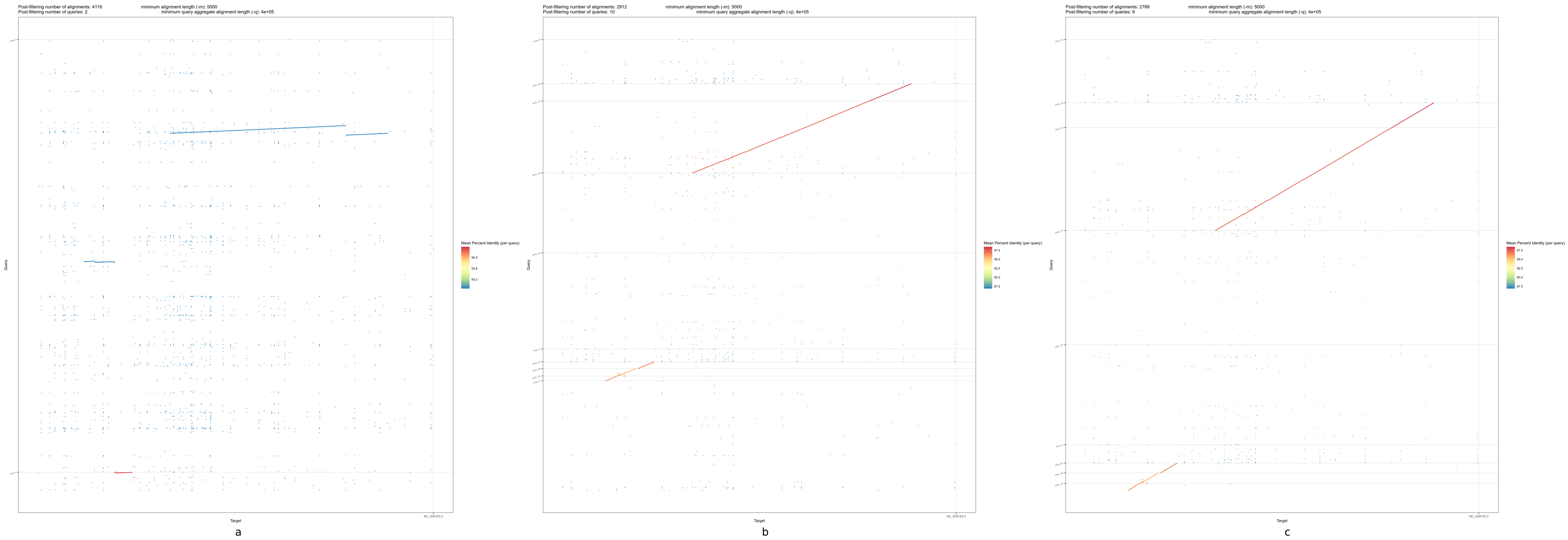

Figure S32: The dot plots of re-assembled scaffolds and the donkey reference chromosome 10 using different cutoffs. (a) cutoff=0.1; (b) cutoff=0.2; (c)cutoff=0.3.

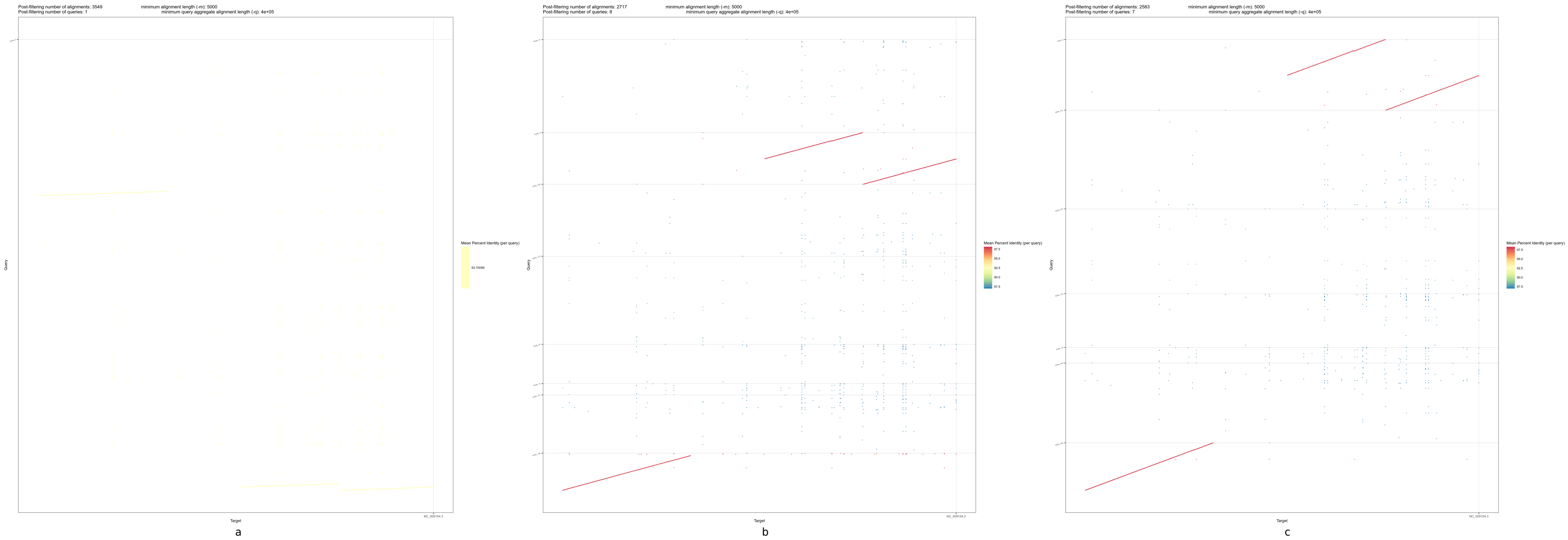

Figure S33: The dot plots of re-assembled scaffolds and the donkey reference chromosome 11 using different cutoffs. (a) cutoff=0.1; (b) cutoff=0.2; (c)cutoff=0.3.

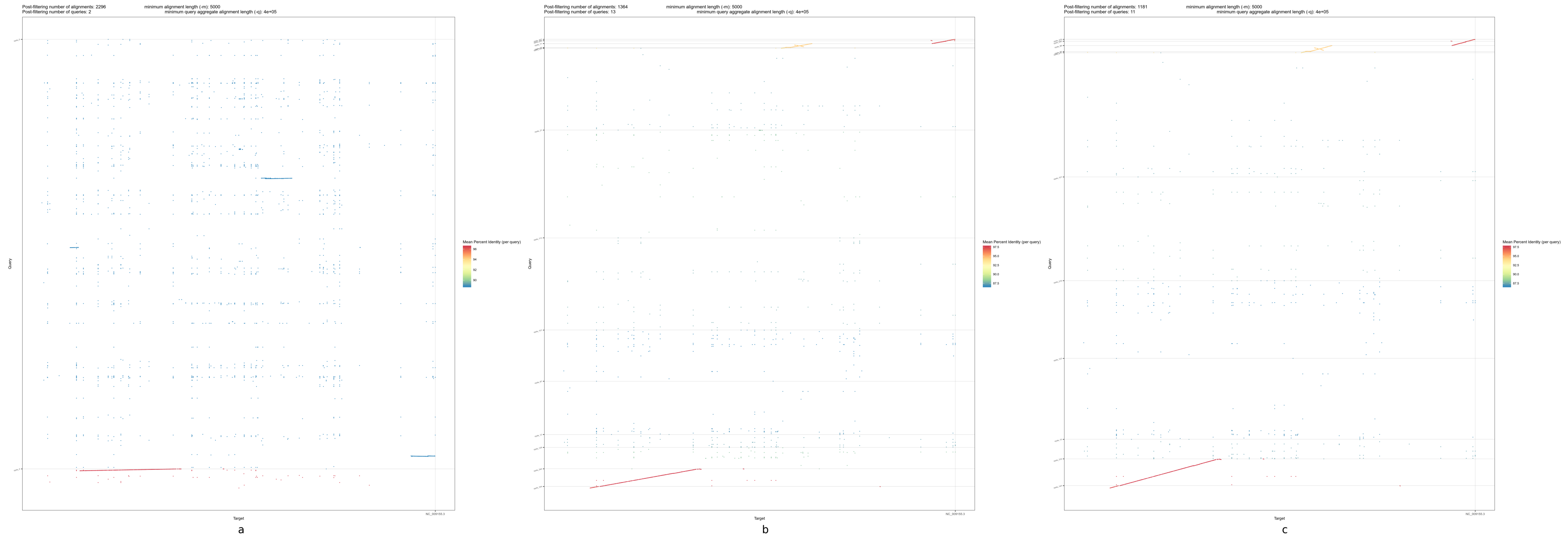

Figure S34: The dot plots of re-assembled scaffolds and the donkey reference chromosome 12 using different cutoffs. (a) cutoff=0.1; (b) cutoff=0.2; (c)cutoff=0.3.

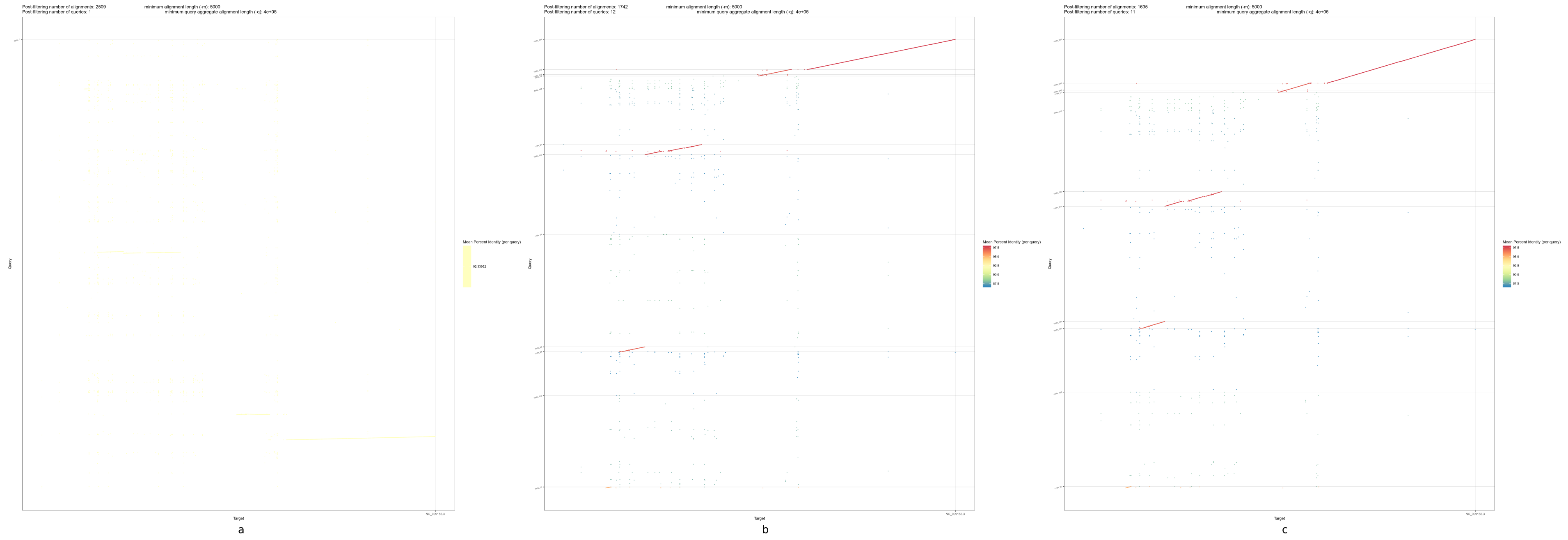

Figure S35: The dot plots of re-assembled scaffolds and the donkey reference chromosome 13 using different cutoffs. (a) cutoff=0.1; (b) cutoff=0.2; (c)cutoff=0.3.

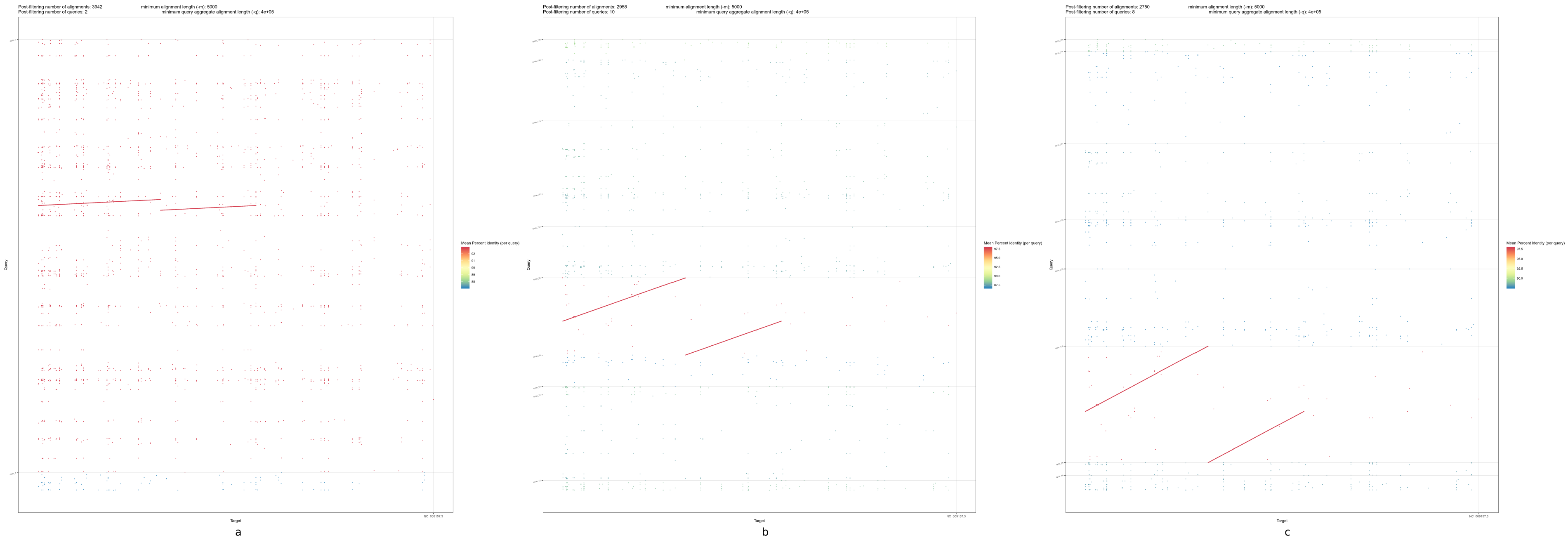

Figure S36: The dot plots of re-assembled scaffolds and the donkey reference chromosome 14 using different cutoffs. (a) cutoff=0.1; (b) cutoff=0.2; (c)cutoff=0.3.

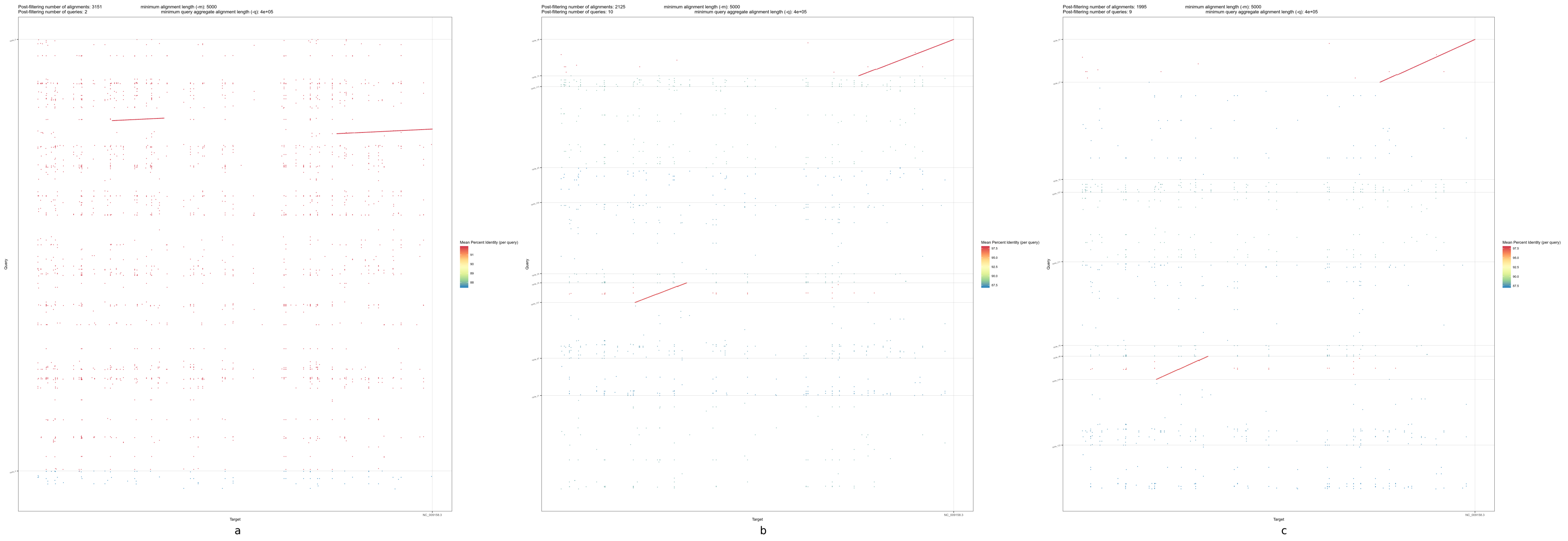

Figure S37: The dot plots of re-assembled scaffolds and the donkey reference chromosome 15 using different cutoffs. (a) cutoff=0.1; (b) cutoff=0.2; (c)cutoff=0.3.

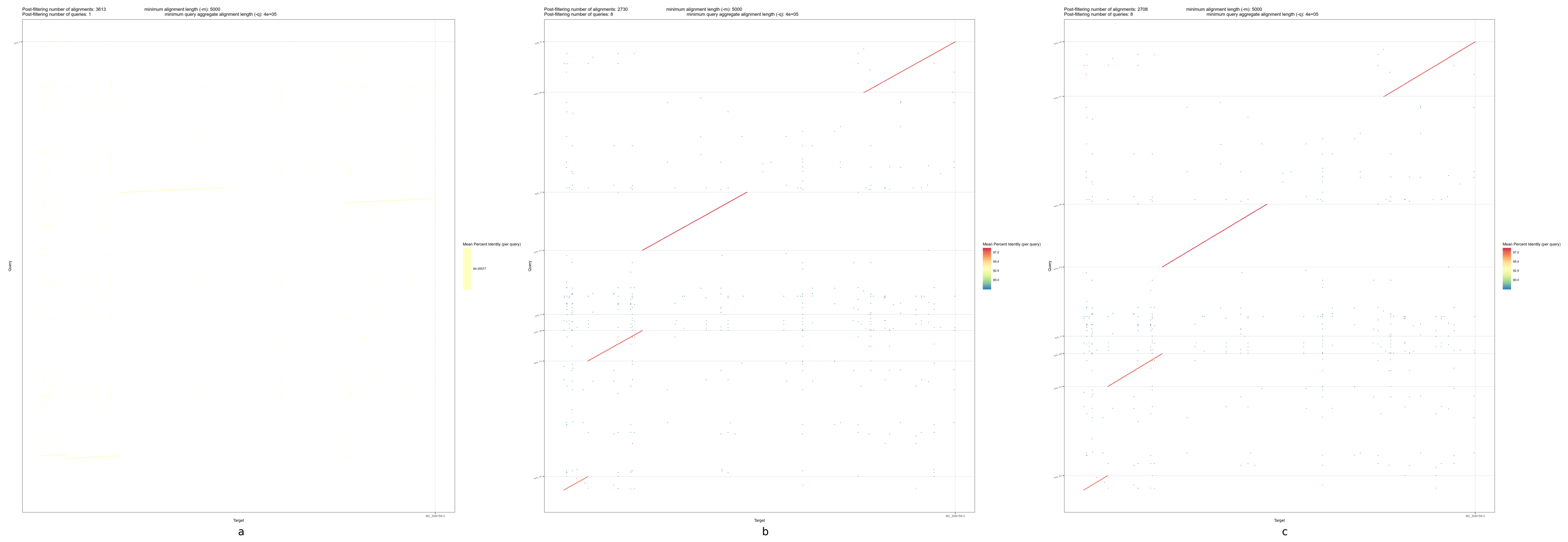

Figure S38: The dot plots of re-assembled scaffolds and the donkey reference chromosome 16 using different cutoffs. (a) cutoff=0.1; (b) cutoff=0.2; (c)cutoff=0.3.

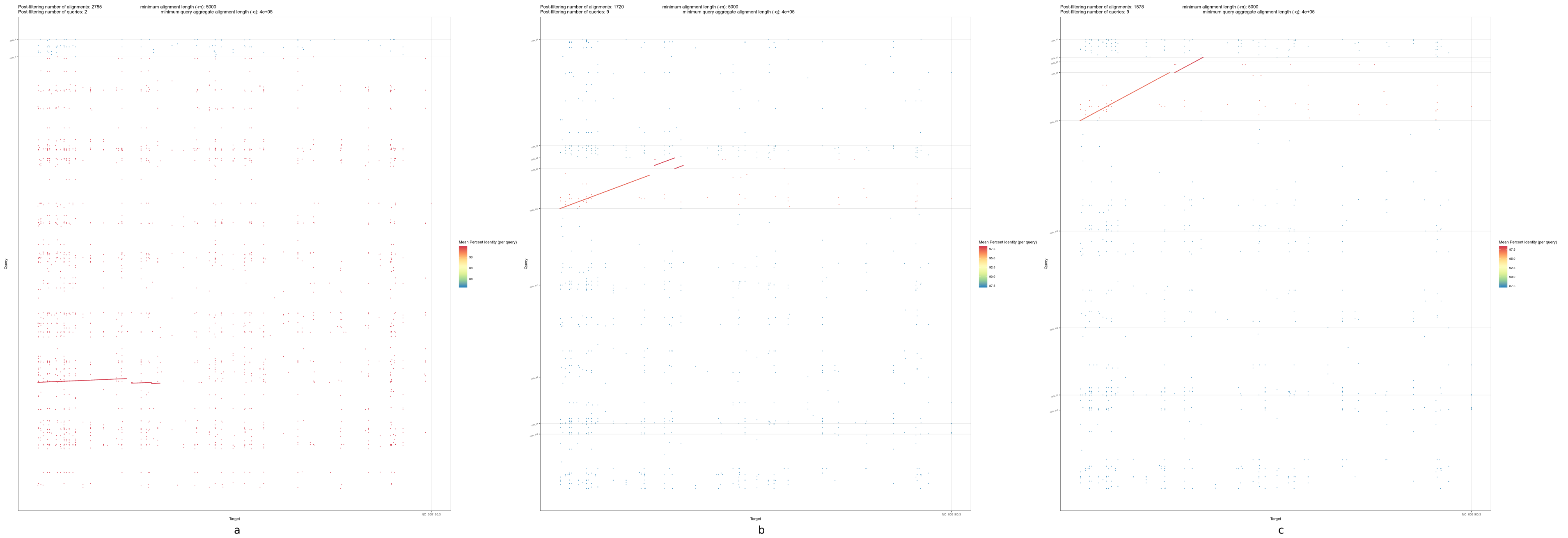

Figure S39: The dot plots of re-assembled scaffolds and the donkey reference chromosome 17 using different cutoffs. (a) cutoff=0.1; (b) cutoff=0.2; (c)cutoff=0.3.

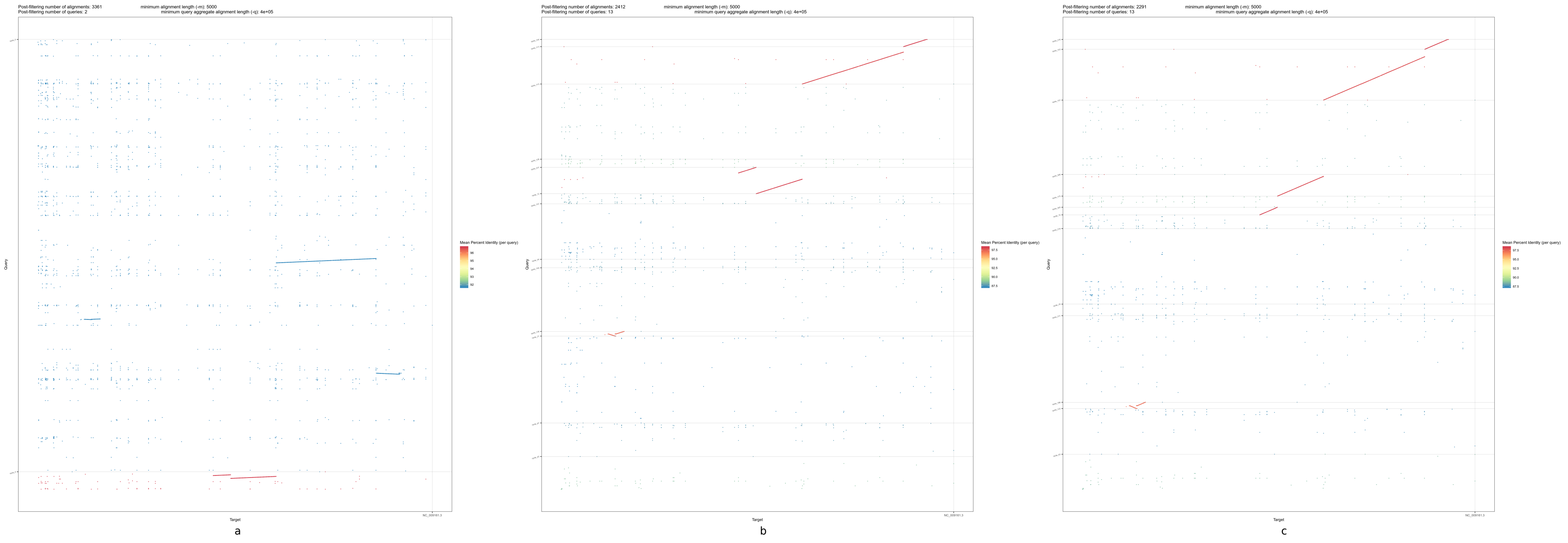

Figure S40: The dot plots of re-assembled scaffolds and the donkey reference chromosome 18 using different cutoffs. (a) cutoff=0.1; (b) cutoff=0.2; (c)cutoff=0.3.

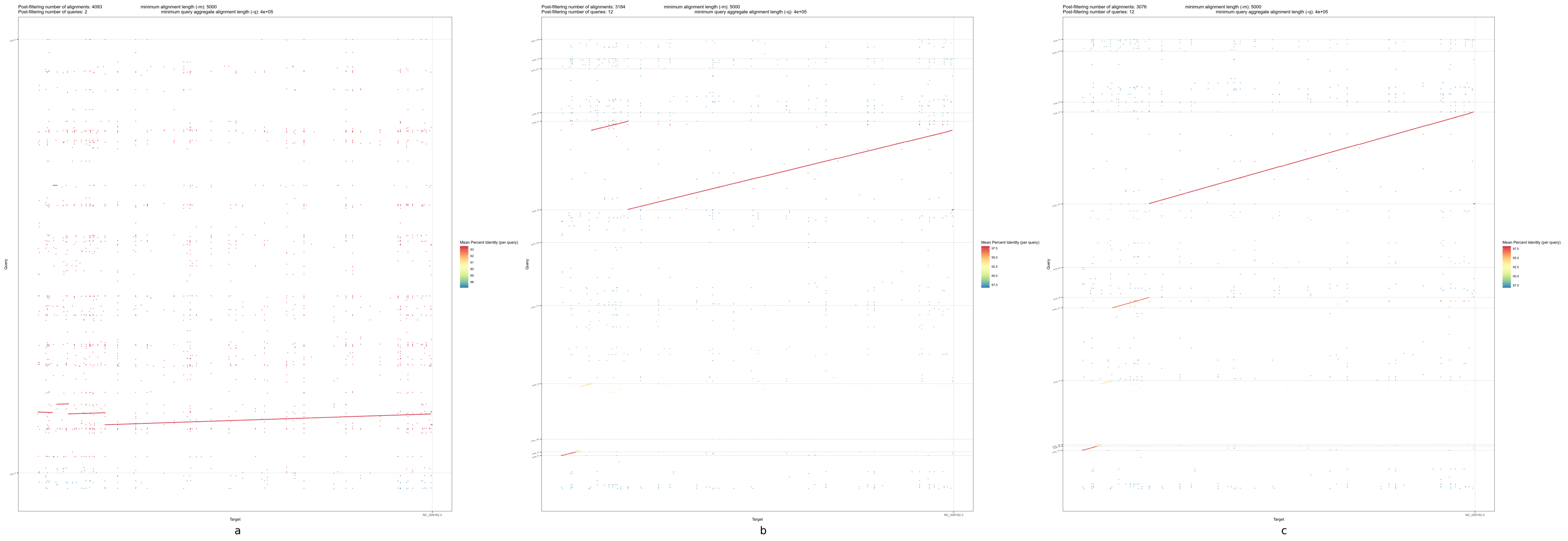

Figure S41: The dot plots of re-assembled scaffolds and the donkey reference chromosome 19 using different cutoffs. (a) cutoff=0.1; (b) cutoff=0.2; (c)cutoff=0.3.

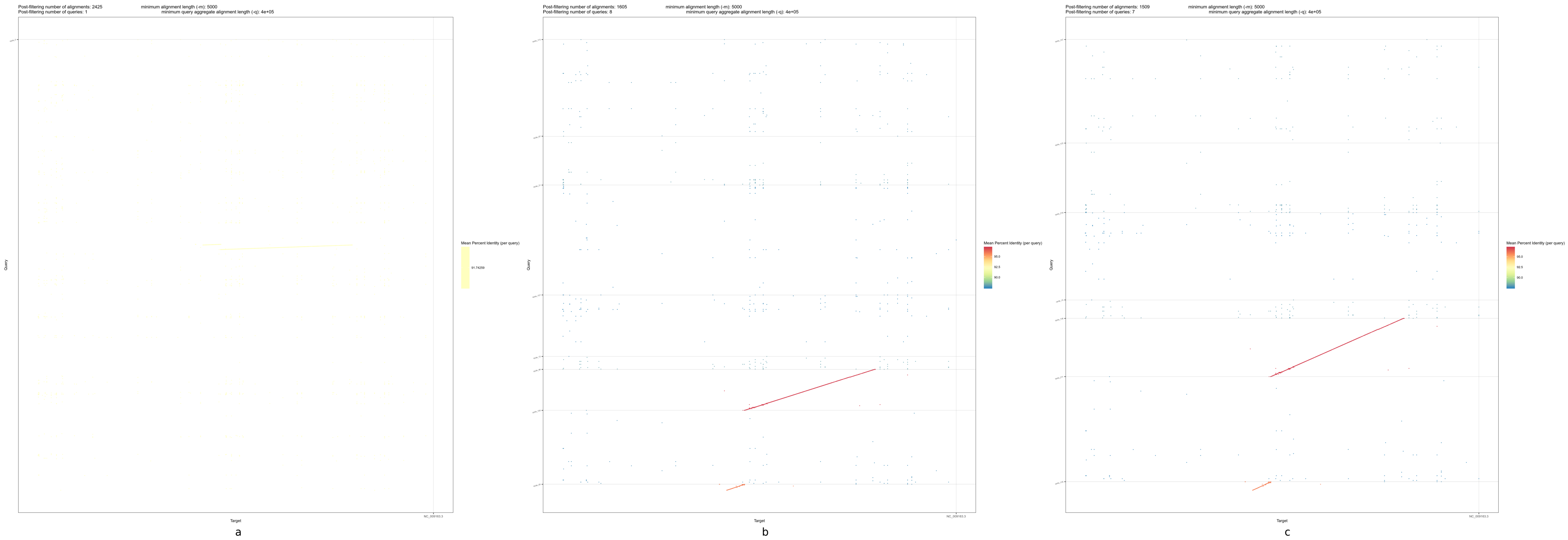

Figure S42: The dot plots of re-assembled scaffolds and the donkey reference chromosome 20 using different cutoffs. (a) cutoff=0.1; (b) cutoff=0.2; (c)cutoff=0.3.

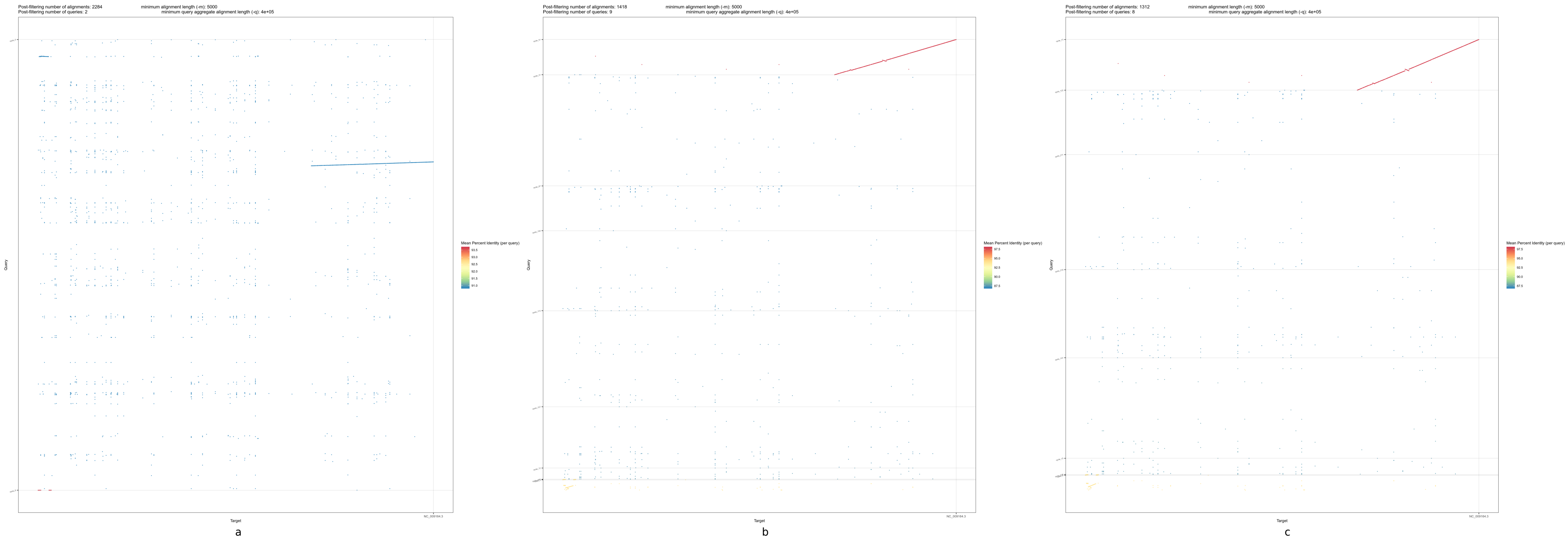

Figure S43: The dot plots of re-assembled scaffolds and the donkey reference chromosome 21 using different cutoffs. (a) cutoff=0.1; (b) cutoff=0.2; (c)cutoff=0.3.

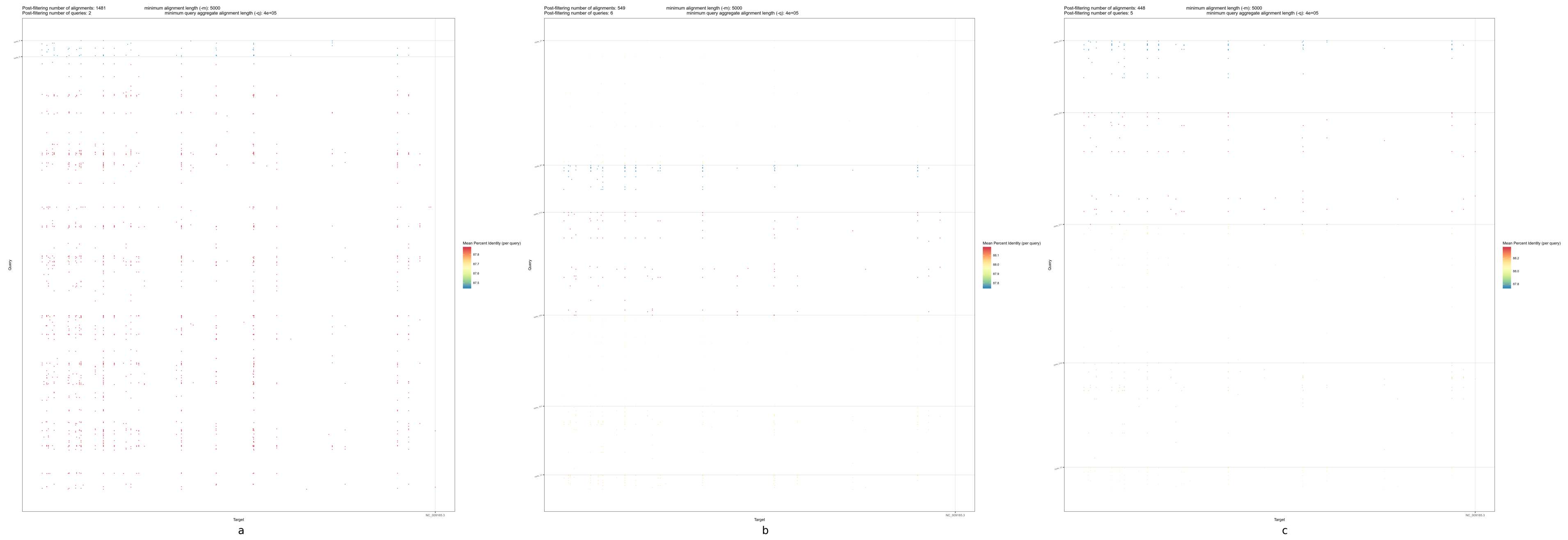

Figure S44: The dot plots of re-assembled scaffolds and the donkey reference chromosome 22 using different cutoffs. (a) cutoff=0.1; (b) cutoff=0.2; (c)cutoff=0.3.

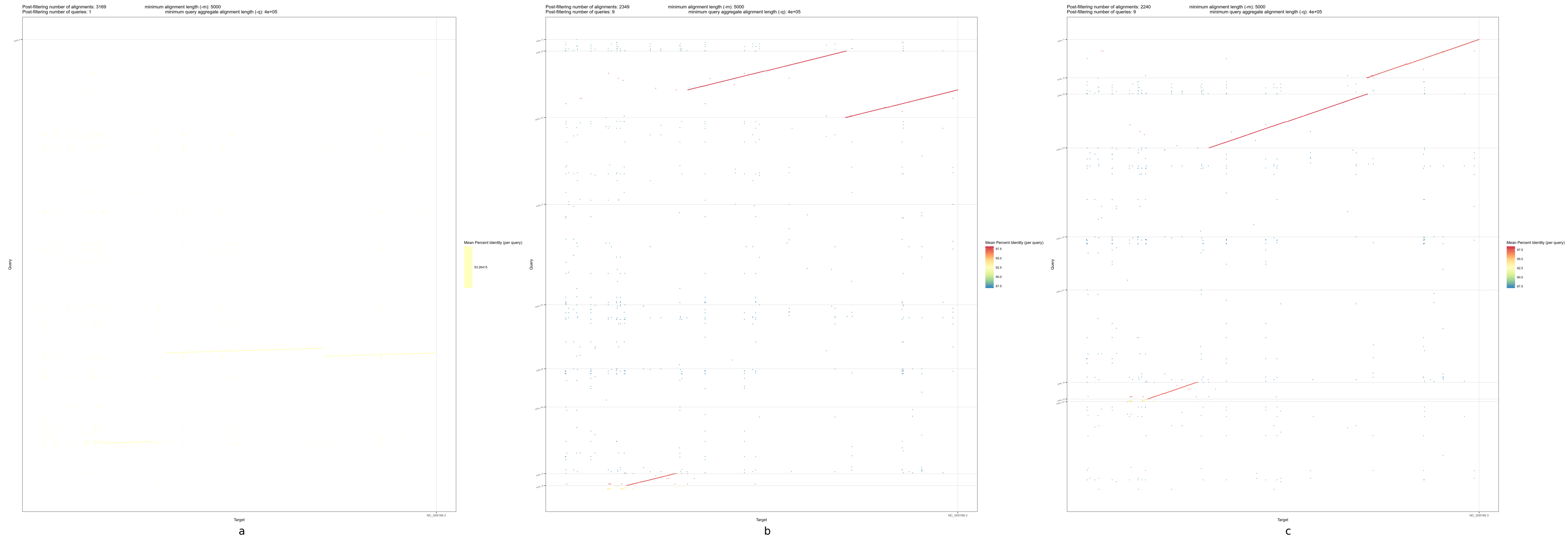

Figure S45: The dot plots of re-assembled scaffolds and the donkey reference chromosome 23 using different cutoffs. (a) cutoff=0.1; (b) cutoff=0.2; (c)cutoff=0.3.

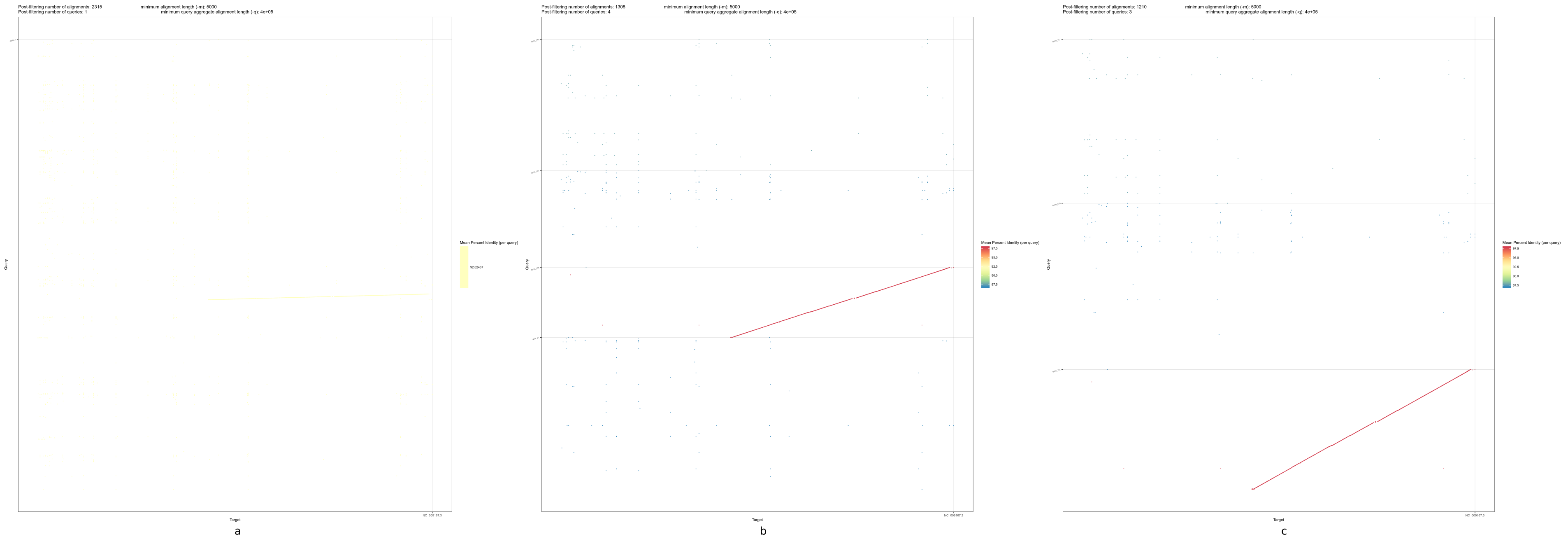

Figure S46: The dot plots of re-assembled scaffolds and the donkey reference chromosome 24 using different cutoffs. (a) cutoff=0.1; (b) cutoff=0.2; (c)cutoff=0.3.

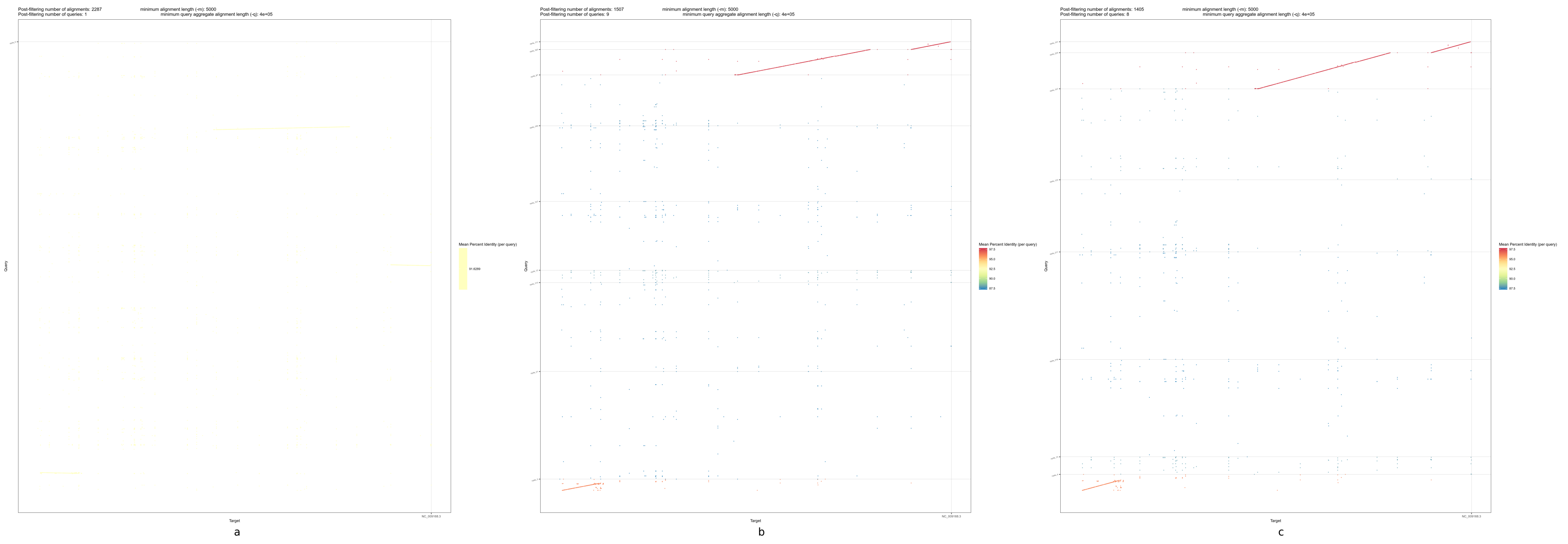

Figure S47: The dot plots of re-assembled scaffolds and the donkey reference chromosome 25 using different cutoffs. (a) cutoff=0.1; (b) cutoff=0.2; (c)cutoff=0.3.

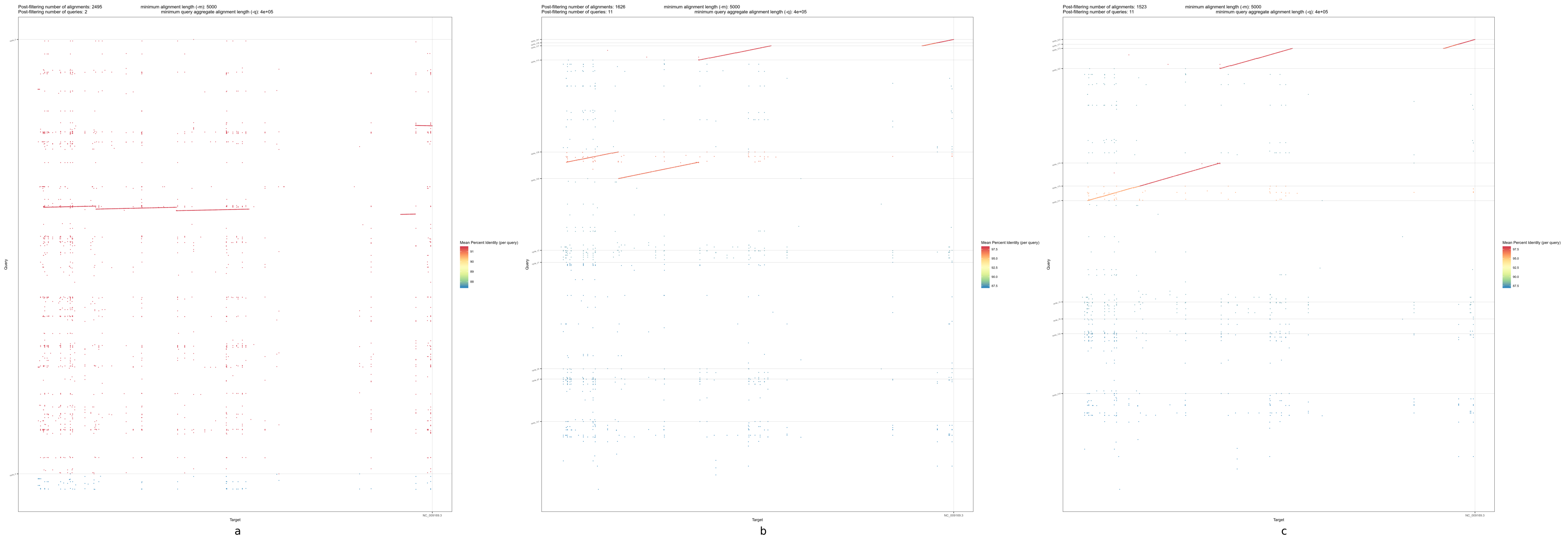

Figure S48: The dot plots of re-assembled scaffolds and the donkey reference chromosome 26 using different cutoffs. (a) cutoff=0.1; (b) cutoff=0.2; (c)cutoff=0.3.

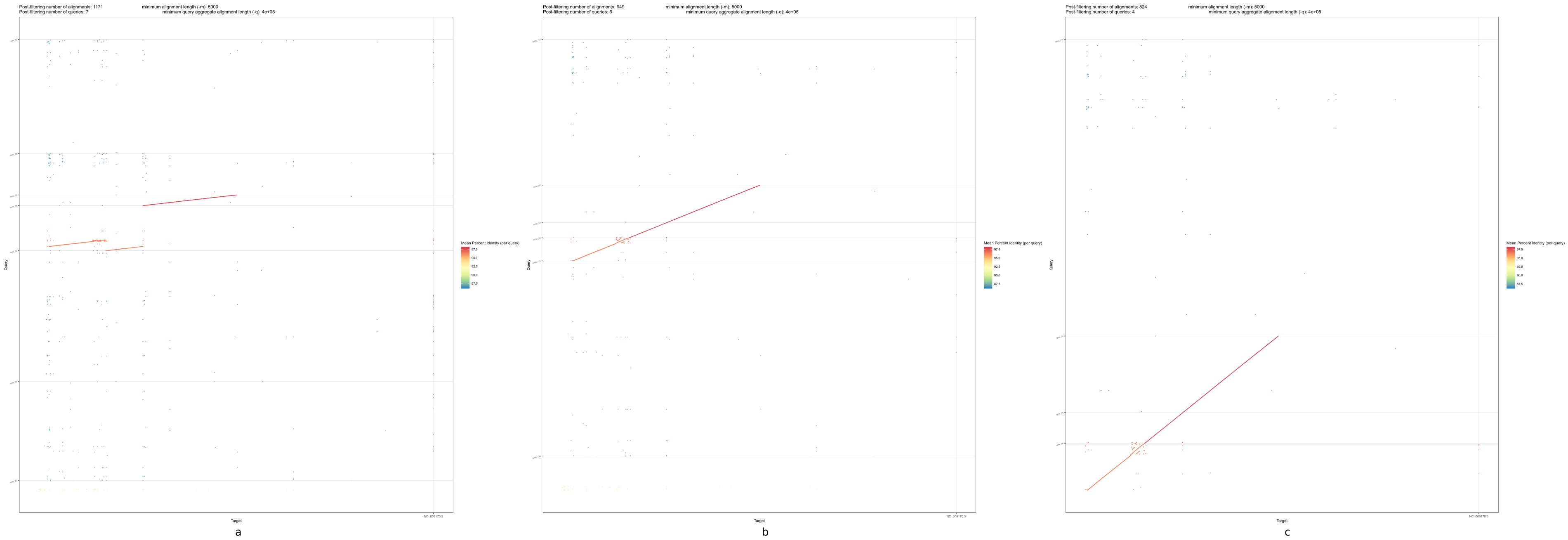

Figure S49: The dot plots of re-assembled scaffolds and the donkey reference chromosome 27 using different cutoffs. (a) cutoff=0.1; (b) cutoff=0.2; (c)cutoff=0.3.

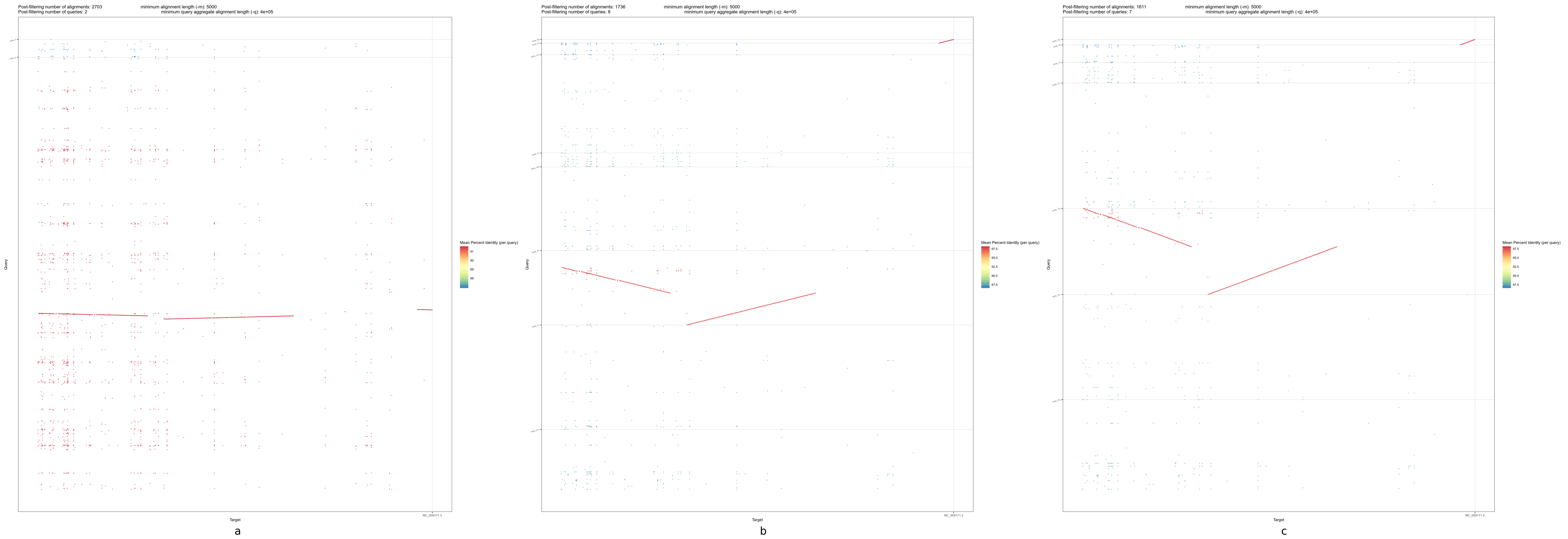

Figure S50: The dot plots of re-assembled scaffolds and the donkey reference chromosome 28 using different cutoffs. (a) cutoff=0.1; (b) cutoff=0.2; (c)cutoff=0.3.

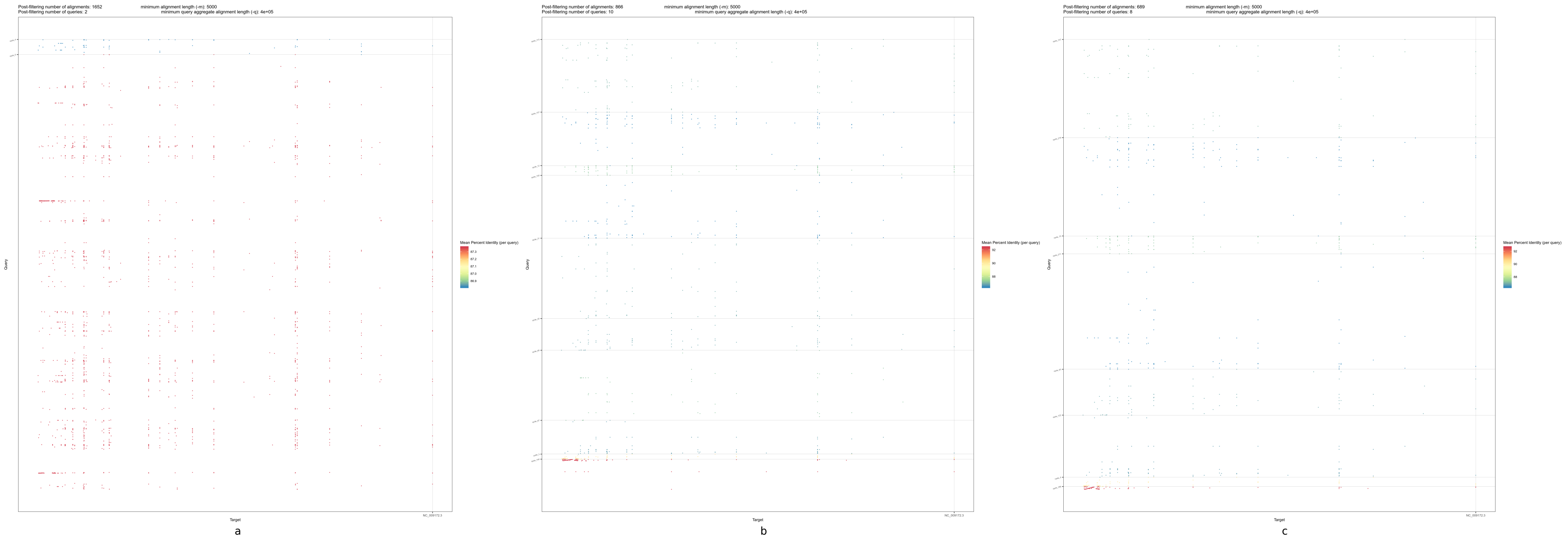

Figure S51: The dot plots of re-assembled scaffolds and the donkey reference chromosome 29 using different cutoffs. (a) cutoff=0.1; (b) cutoff=0.2; (c)cutoff=0.3.

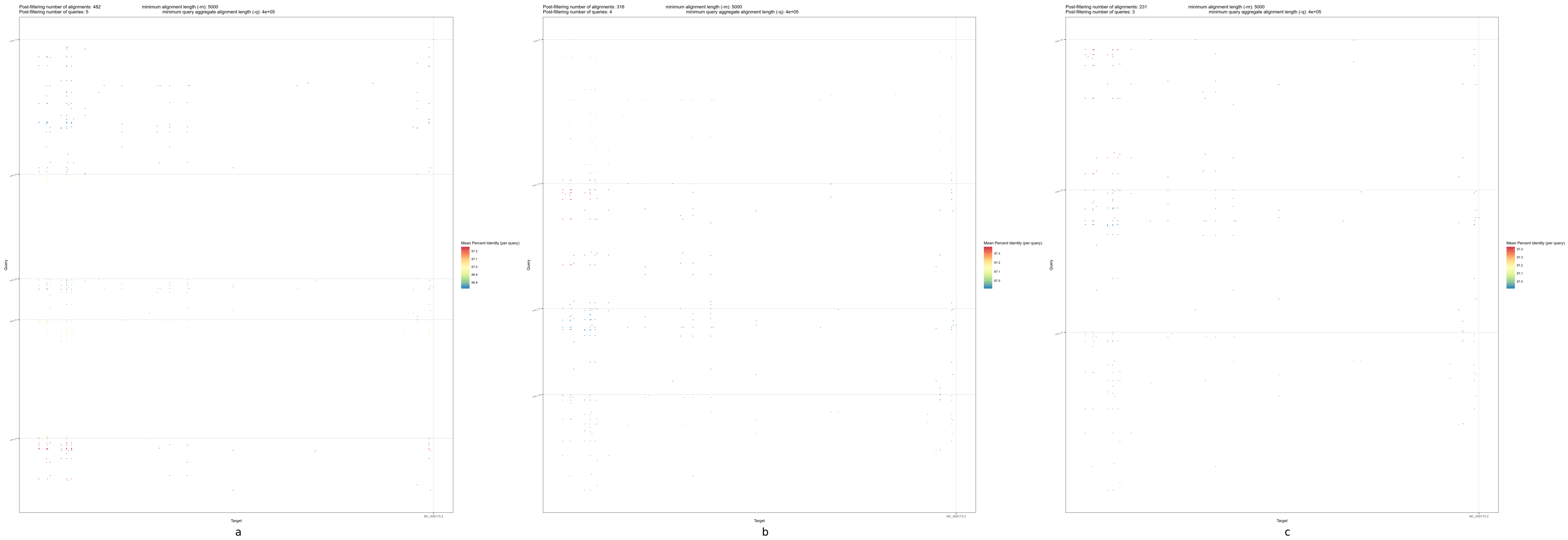

Figure S52: The dot plots of re-assembled scaffolds and the donkey reference chromosome 30 using different cutoffs. (a) cutoff=0.1; (b) cutoff=0.2; (c)cutoff=0.3.

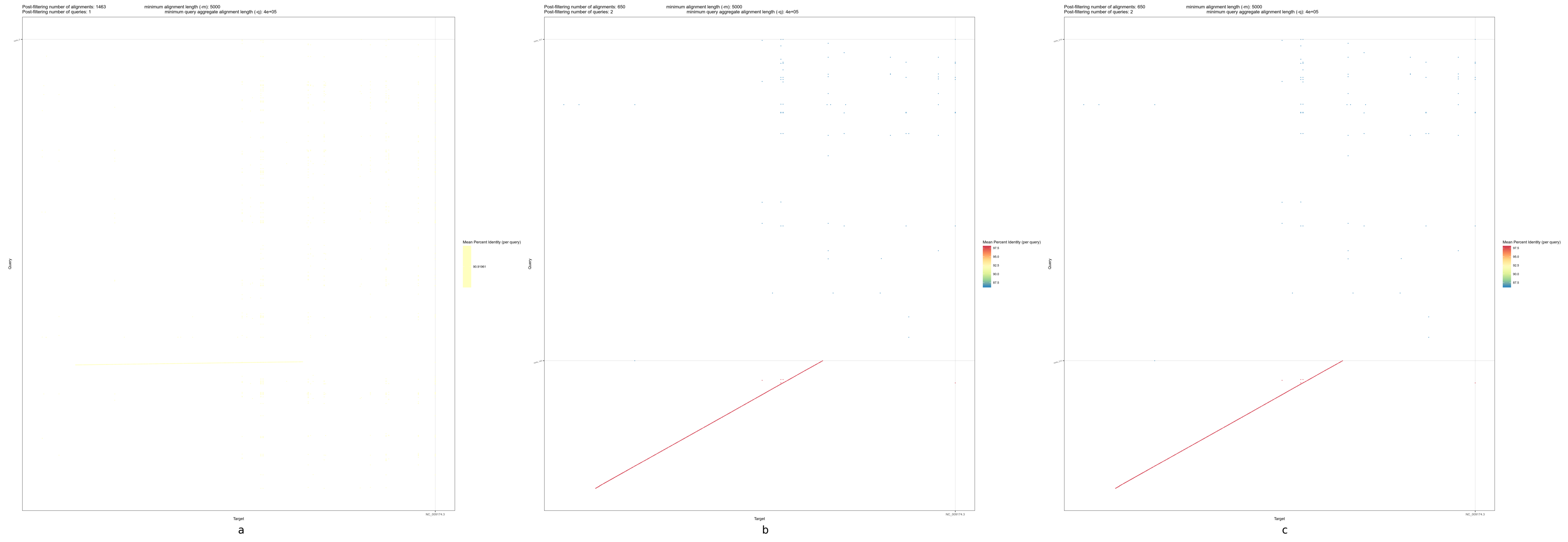

Figure S53: The dot plots of re-assembled scaffolds and the donkey reference chromosome 31 using different cutoffs. (a) cutoff=0.1; (b) cutoff=0.2; (c)cutoff=0.3.

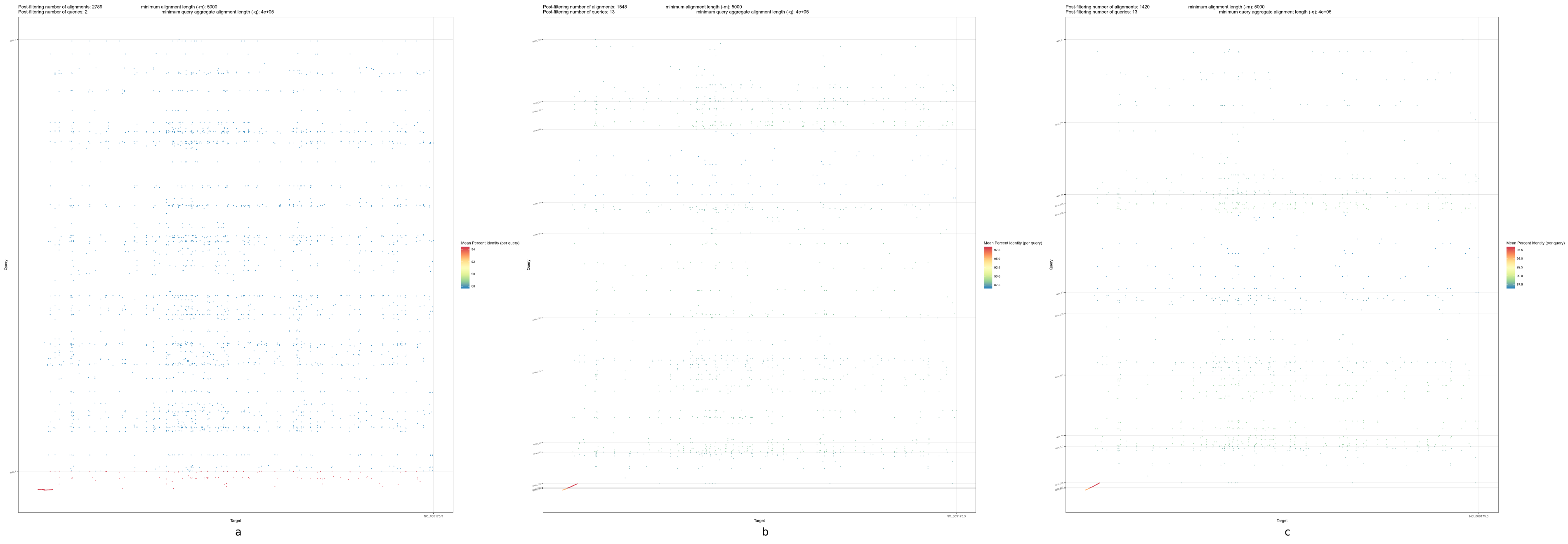

Figure S54: The dot plots of re-assembled scaffolds and the donkey reference chromosome X using different cutoffs. (a) cutoff=0.1; (b) cutoff=0.2; (c)cutoff=0.3.
